# Bibliometric analysis of Naunyn–Schmiedeberg’s Archives of Pharmacology (1947–1974)

**DOI:** 10.1007/s00210-024-03078-8

**Published:** 2024-04-23

**Authors:** Mert Erkan Basol, Roland Seifert

**Affiliations:** Institute of Pharmacology, Hannover Medical School, 30625 Hannover, Germany

**Keywords:** Bibliometric analysis, Naunyn–Schmiedeberg’s Archives of Pharmacology, SpringerLink, Publication trends, Citation analysis, Language in scientific publication, Historical analysis of journals

## Abstract

Naunyn–Schmiedeberg’s Archives of Pharmacology is the oldest pharmacological journal, founded in 1873. This bibliometric analysis examines the pivotal transformations within Naunyn–Schmiedeberg’s Archives of Pharmacology from 1947 to 1974, identifying significant shifts from a national focus to a period of extensive internationalization and English-language adoption. Employing Python and Beautiful Soup for data extraction from SpringerLink, the study maps the journal’s trajectory through post-World War II development, highlighting the decline in publication rates due to its initial emphasis on German-language articles predominantly from Germany. The transition towards English publications in the late 1960s is marked as a turning point, catalyzing an increase in global citations, publications, and recognition. This period witnesses the journal broadening its scientific horizon, with a notable emphasis on the cholinergic, adrenergic, and dopaminergic systems, reflecting their central role in the journal’s scientific discourse and citation prominence. The analysis demonstrates how shifting to English for academic publishing played a crucial role in revitalizing the journal’s impact and visibility on the international stage.

**Supplementary information:**

The online version contains supplementary material available at 10.1007/s00210-024-03078-8.

## Introduction

In 1873, Bernhard Naunyn, Oswald Schmiederberg, and Edwin Klebs founded the “Archiv für experimentelle Pathologie und Pharmakologie,” which has evolved into the Naunyn–Schmiedeberg’s Archives of Pharmacology. Their collaboration in Dorpat, where Naunyn and Schmiedeberg served as professors, was foundational in creating a journal that would integrate the fields of pathology and pharmacology. Schmiedeberg is particularly noted as a pioneer of experimental pharmacology, with a lasting influence that extends throughout the global pharmacological community (Starke 1998).

Quickly after its founding, Naunyn-Schmiedeberg’s Archives of Pharmacology was recognized as one of the most important journals in its field, being the oldest pharmacological journal. This reputation has been consistently upheld (Koch-Weser and Schechter 1978; Starke 1998). However, the aftermath of the Second World War presented significant challenges, including a 2-year publication hiatus that marked a period of re-evaluation and recovery for the journal (Starke 1998). This era coincided with the emergence of a new international scientific community, from which Germany was initially isolated (Ahlers et al. 2023).

A recent bibliometric analysis (Dats et al. 2023), which primarily examined the early twenty-first century using distinct datasets, did not address the post-war period in detail, leaving a gap in the literature. Despite Klaus Starke’s comprehensive historical overview on the first 125 years of Naunyn–Schmiedeberg’s Archives of Pharmacology (Starke 1998), there is a lack of focused analysis on Naunyn–Schmiedeberg’s Archives of Pharmacology’s evolution in the post-World War II period. This era, critical for the journal’s shift towards internationalization and English publication, significantly boosted its citations and global presence. 

By providing an overview of the journal’s development from the post-war period up to 1974, this paper aims to contribute to this existing gap. To offer insights into Germany’s efforts to reassert its scientific contributions on the global stage, this paper places the bibliometric findings within a historical, political, and scientific context.

## Materials and methods

### Extraction process for publication data

**Fig. 1:**
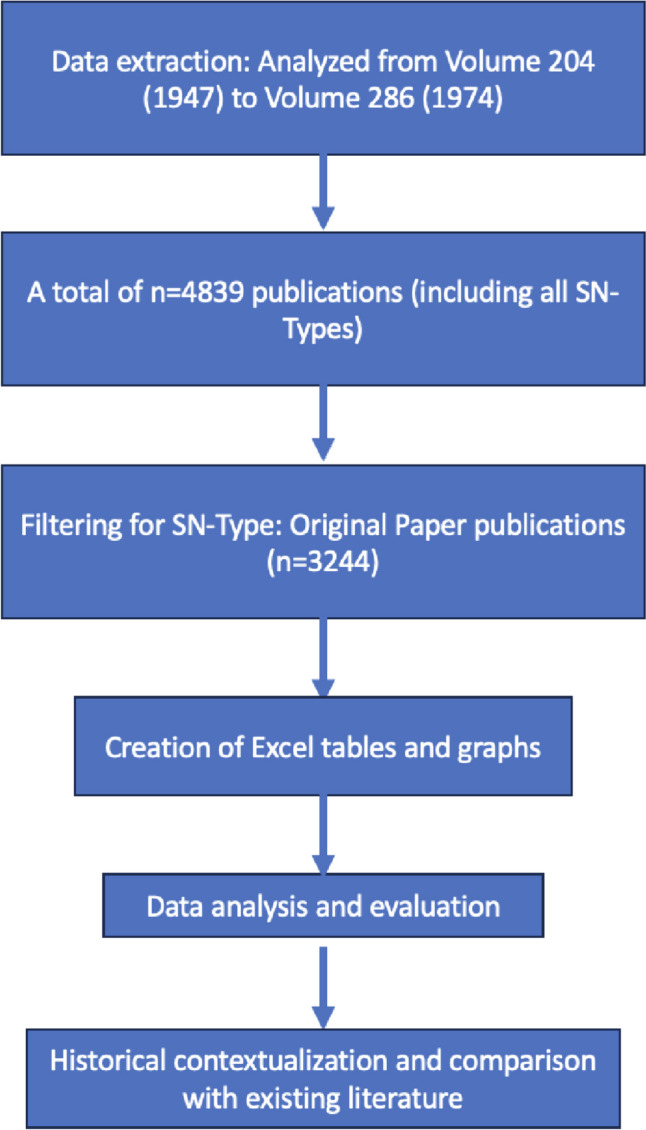
Flowchart representation of the analysis procedure

The bibliometric analysis of Naunyn–Schmiedeberg’s Archives of Pharmacology was conducted using Python and Beautiful Soup, focusing on extracting publication details from the official SpringerLink website (https://link.springer.com/journal/210/volumes-and-issues; Python Software Foundation 2021; Richardson 2021; Springer Link 2024). This methodological choice was driven by the need for a comprehensive and automated approach to data collection over a significant historical span, specifically targeting volumes 204 (1947) to 286 (1974) and encompassing a range of publication metrics from a total of 4839 publications identified across 224 cities and 44 countries (Fig. 1). The previously used data extraction using Excel yielded substantial data gaps for the period from 1947 to 1971 (Dats et al. 2023) which were now closed with the comprehensive Phython and Beautiful Soup approach.

### Identification of publication metrics

The extracted data included various publication metrics such as the SN type (original papers, abstracts, short papers, announcements, discussions, editorial notes, erratum, opening speeches, main topics, keynote lectures, short communications, symposiums, DGPT spring meetings, demonstrations), titles, author names (first, second, and last), affiliations, DOI numbers, issue dates and years, volume numbers, and citation counts. Citation numbers were obtained via CrossRef (https://www.crossref.org/about/) which is integrated with the SpringerLink page, ensuring the accuracy of citation data.

**Fig. 2:**
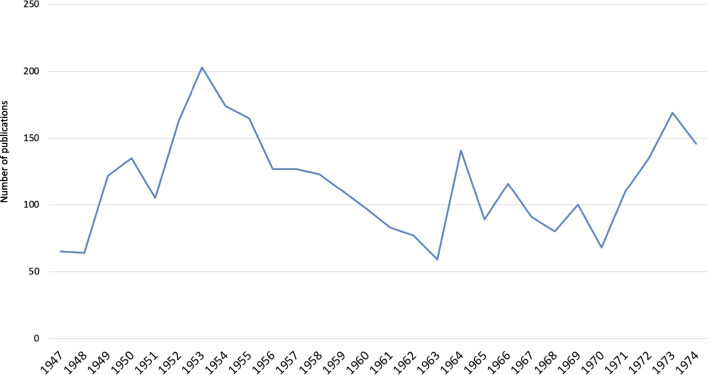
Number of annual publications (Original Papers) from 1947 to 1974

For this analysis, the focus was narrowed to “Original Paper” SN type, resulting in a dataset of 3244 publications (Fig. 1). The Orignal Papers were then analyzed over the years (Fig. 2). These data were then organized into a table using the Pandas library in Python and saved in an Excel (.xlsx) format. To visualize the findings, charts and tables were generated from the Excel spreadsheet.

### Data structuring and accuracy assurance

To validate the accuracy of the extracted data, a Python unit test was conducted. This test compared the data in the Excel table against the original information on the SpringerLink page, ensuring the reliability of the data collection process. This rigorous methodological approach not only underscores the thoroughness of the analysis but also guarantees the credibility of the findings derived from the bibliometric study.

### Language trends in publications

**Fig. 3:**
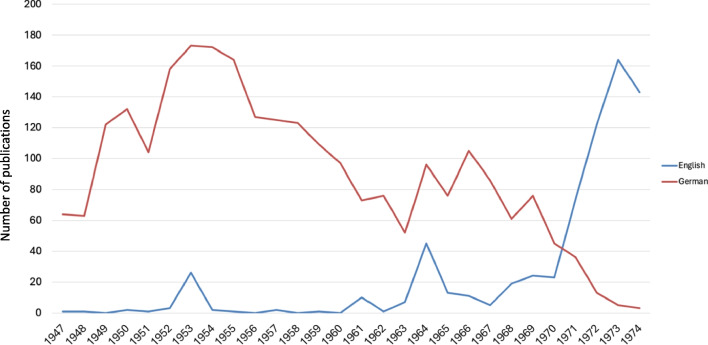
Comparative analysis of publication languages (Original Papers) from 1947 to 1974

Leveraging Python’s langdetect module, the language of each publication (Original Papers) was discerned from the titles once the data had been structured into an Excel spreadsheet. This enabled a longitudinal analysis of the languages used in the publications spanning from 1947 to 1974. It was observed that articles were published in French and Italian on a few occasions—six times and once, respectively. However, to ensure clarity and focus on the visual representation of the data, these instances were omitted from the graphical analysis presented in Fig. 3.

### Citation analysis

**Fig. 4:**
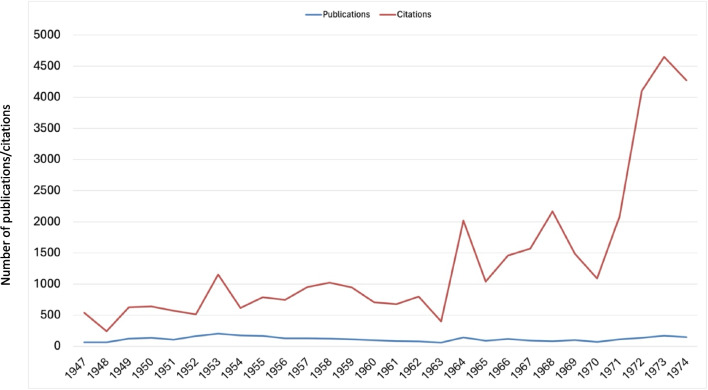
Trend analysis of publications (Original Papers) and citations from 1947 to 1974

For citation analysis, the number of citations for each publication (Original Papers) was retrieved through CrossRef, utilizing Python and Beautiful Soup, as of January 2, 2024. CrossRef, which is updated daily and directly linked to the SpringerLink page, provided a comprehensive view of the citation patterns across the studied period (1947–1974). This dataset facilitated a granular examination of citation behavior over the years, allowing for an in-depth understanding of the factors influencing citation frequency, such as publication number and citation quotient (Fig. 4, Tab. S1).

### Analysis of the 100 most-cited articles

**Fig. 5:**
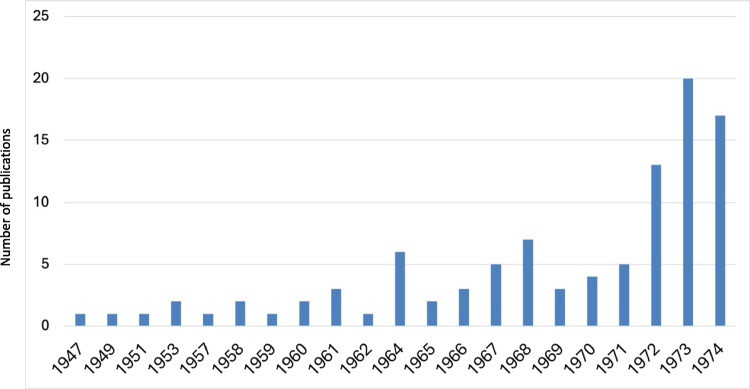
Distribution of the 100 most cited articles (Original Papers) over time from 1947 to 1974

**Fig. 6:**
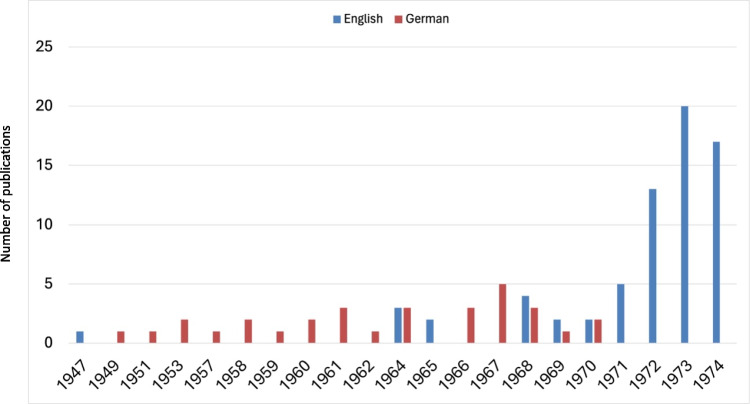
Linguistic distribution of the 100 most cited articles (Original Papers) from 1947 to 1974

**Fig. 7:**
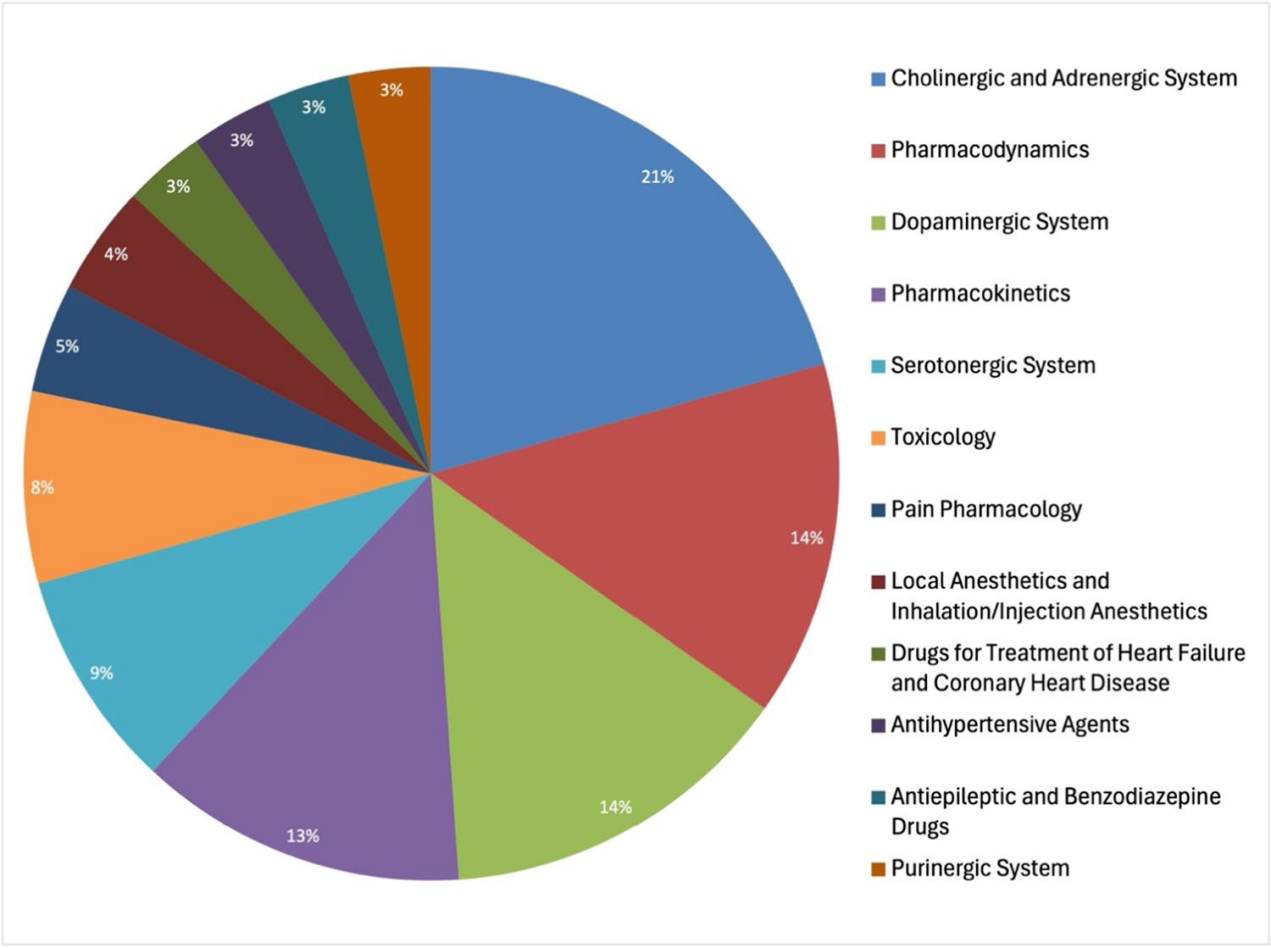
Topic distribution of the 100 most cited articles (Original Papers)

**Fig. 8:**
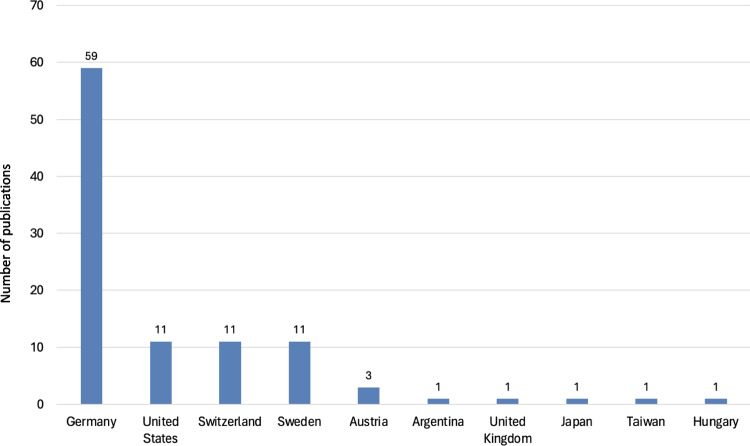
Comparative distribution of the top 100 most-cited research articles (Original Papers) by country of origin

Further analysis was conducted on the 100 most-cited articles (Original Papers) within the specified timeframe, offering insights into the reasons behind their frequent citation. This examination included the years of publication (Fig. 5), impact of the publication language (Fig. 6), and the thematic focus (Fig. 7), on their citation rates. Additionally, the geographic origins of these highly cited articles were analyzed (Fig. 8), with the findings summarized in a detailed table (Table 1).

### Topics

For simplicity, the entire dataset, excluding publication titles, was translated into English. The titles themselves are crucial for identifying the thematic trends and author focus within the period analyzed. To systematically categorize these themes, the study utilized the textbook “Basiswissen Pharmakologie” as a framework for grouping (Seifert 2018). The textbook’s main topics provided a structured basis, with the addition of “Purinergic system,” “Substance P,” and “Toxicology” to encompass frequently occurring themes not originally listed. This thematic grouping facilitated trend analyses and the exploration of topic-related dynamics over time (Fig. S2). Furthermore, a pie chart analysis (Fig. 7) highlights the distribution of topics within the 100 most cited articles, offering insights into the most influential research areas.

### Authors

**Fig. 9:**
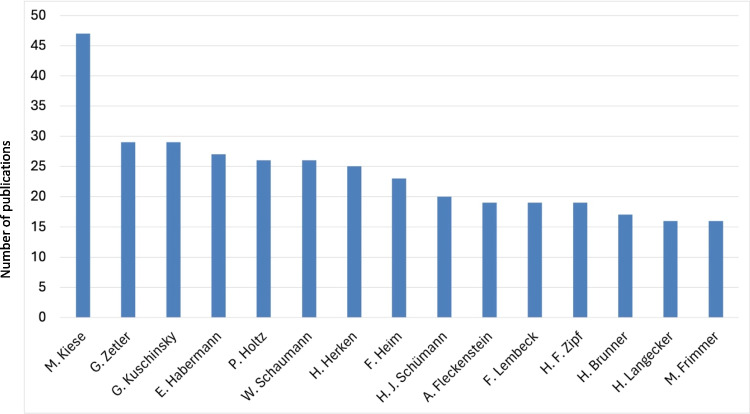
Leading 15 authors based on publications (Original Papers) from 1947 to 1974

To dissect the authorship patterns within Naunyn–Schmiedeberg’s Archives of Pharmacology from 1947 to 1974, a comprehensive analysis was conducted not only on the first authors but also on the second and last authors of the papers. This methodical examination uncovered distinct trends in publication frequency among authors, culminating in the identification of the top 15 contributors to the journal during this period (Fig. 9). An in-depth analysis of the top five authors was subsequently carried out, focusing on their affiliations, thematic interests, and rates of publication (see Tab. S6).

### Geographical analysis of publication origins

Additionally, the geographical origins of the publications (Original Papers) were analyzed. City and country information extracted from the SpringerLink page was anglicized for consistency. A Python script facilitated the classification of countries into their respective continents, enabling a focused analysis of the geographical distribution of the research contributions. This analysis was narrowed down to the top five contributing countries, which together accounted for 97% of the journal’s publications between 1947 and 1974. The cities leading in publication volume were highlighted in a bar chart, providing a visual representation of the geographical trends in research output (Figs. S3, S4 and S5).

Furthermore, to offer a granular view of the contributions within Germany, a heat map was created using Plotly in Python. This visual tool effectively showcased the density of publications across German cities, offering insightful perspectives on the regional distribution of research within the country. This layered approach, combining authorship trends with geographical analysis, provides a nuanced understanding of the journal’s contributions to the pharmacologcial community over the specified period.

## Results and discussion

### Publication activity

Following World War II, Naunyn–Schmiedeberg’s Archives of Pharmacology faced a publication halt for 2 years, resuming in February 1947 with its first post-war volume (204), delayed due to US intelligence control oversight (Starke 1998; Herken 1999). Initially, the journal’s productivity was low, publishing around 70 articles each in 1947 and 1948, a direct consequence of war-induced destruction of academic facilities and the emigration of scientists during the Nazi regime, hindering immediate post-war scientific activities (Philippu 2004–2021; Löffelholz 2011; Weise-Pötschke 2019; Heinsohn and Nicolaysen 2021; Dats et al. 2023; Hattori and Seifert 2023; Fig. 2).

By 1949, publication numbers surged to 122, reflecting the release of wartime research (Dats et al. 2023). This growth continued, notably doubling around 1951 and 1953, partly due to celebrating pharmacologist Otto Loewi’s 80th birthday (Starke 1998). Despite a peak in 1964 honoring Otto Krayer’s 65th birthday, the journal experienced a gradual decline in the number of publications until 1970, influenced by the preference for publishing in higher-impact English-language journals (Zehetbauer et al. 2022; Dats et al. 2023; Gzoyan et al. 2023).

The shift towards English, essential for international scientific communication, led to the marginalization of German-language journals (Gzoyan et al. 2023). Recognizing the need for internationalization, the journal’s editors initiated significant changes in the late 1960s, including the internationalization of the editorial board and the transition to publishing in English, culminating in the journal’s name change in 1971 and the mandate for English publications from 1973 onwards. These measures revitalized the journal, as evidenced by a 149% increase in publications from 1970 to 1973, stabilizing its contribution to the international research community thereafter (Starke 1998; Dats et al. 2023; Hattori and Seifert 2023; Fig. 2).

### Language

Naunyn–Schmiedeberg’s Archives of Pharmacology predominantly featured German-language publications until the late 1960s, reflecting its national orientation (Fig. 3). This linguistic homogeneity meant its research largely remained within German-speaking circles, limiting international engagement and recognition (Bajerski 2011). Analysis of publication trends (Fig. 2) against the language of publication (Fig. 3) reveals a parallel between the overall publication volume and German-language articles up to 1970. Notably, spikes in English publications in 1953 and 1964 corresponded with contributions from the USA, including works dedicated to emigrant pharmacologists Otto Loewi and Otto Krayer, highlighting brief periods of international collaboration (Starke 1998).

Loewi, a former student of Oswald Schmiedeberg, fled Nazi Germany in 1938, eventually settling in the USA at New York University (Bettendorf 1995; Philippu 2004–2021; McCoy and Tan 2014). Similarly, Krayer, who refused a position vacated under Nazi policies, led Harvard University’s pharmacology institute from 1939 to 1966 (Starke 1998; Philippu 2004–2021; Rubin 2014). The contributions of Loewi and Krayer underscore the journal’s intermittent international reach.

The sharp increase in English publications from 1970 onwards (Fig. 3) reflects editorial efforts to promote English, transitioning from five English articles in 1967 to 164 by 1973, with a corresponding decline in German articles. This shift was instrumental in repositioning the journal within the global scientific community (Starke 1998; Francisco 2015; Hattori and Seifert 2023). Post-WWII, Germany’s diminished academic stature and UNESCO’s advocacy for multilingualism in scientific publishing prompted questions about delayed internationalization efforts. The reluctance of the journal’s publisher, Dr. Springer, to adopt multilingualism until the late 1960s contributed to this lag (DGPT archive in Göttingen 01.09.1949).

The ascendancy of English as the primary scientific language post-1970s highlights a broader shift towards internationalization in German science, necessitated by a globalizing research landscape (Winkmann et al. 2002; Heinsohn and Nicolaysen 2021). The debate over the late adoption of English in German journals touches on the broader discourse around multilingualism’s role in ensuring high-quality, globally communicable science (Tardy 2004; Billings 2015; Davydova 2020). The imposition of English as the lingua franca raises ethical questions about linguistic equity and the potential marginalization of non-English speakers (Phillipson 2009; O’Neil 2018). Moreover, the dominance of English reflects colonial legacies, risking the devaluation of other languages and the sidelining of non-English scientific contributions (Phillipson 2009; Ahn et al. 2017). The international neglect of non-English journals, resulting in fewer citations and recognition, underscores the challenges of linguistic isolation in the global scientific community (Phillipson 2009; Gzoyan et al. 2023).

### Citations

While high publication volumes post-1970s indicate increased scientific productivity, assessing the international recognition of Naunyn–Schmiedeberg’s Archives of Pharmacology and its articles requires examining citation patterns (Francisco 2015). Figure 4 presents annual publication counts alongside total citations from 1947 to 1974, calculating an average citation rate per article. This metric reveals a gradual increase in citation impact, with a notable surge from 1963 onwards. Specifically, the citation quotient jumped significantly in the early 1970s, from 17.9 in 1971 to 29.4 in 1972, compared to 7.3 in 1947 and 28.3 in 1974 (see Fig. S1). This trend suggests the journal’s internationalization efforts in the 1970s significantly bolstered its standing within the global research community (Starke 1998; Francisco 2015).

Although high citation rates and impact factors (IFs) are commonly associated with a journal’s academic prestige, IFs have been critiqued as an imperfect measure for evaluating journal quality (Seglen 1998). Nonetheless, citations do play a role in enhancing a publication’s international visibility, with English-language articles typically receiving more citations than non-English papers. This advantage shows that publishing in English can significantly benefit both journals and authors in terms of both international and national recognition (Vinther and Rosenberg 2012). The internationalization measures of the 1970s, therefore, not only enhanced the visibility of Naunyn–Schmiedeberg’s Archives of Pharmacology but also contributed to a broader acknowledgment of the work published within its pages (Starke 1998; Francisco 2015).

### 100 most quoted original papers

Citation behavior significantly impacts both journals and their authors, as high citation counts are often associated with scientific prominence and success (Seglen 1998; Francisco 2015). A detailed bibliometric analysis of the 100 most-cited publications (Original Papers) within Naunyn–Schmiedeberg’s Archives of Pharmacology highlights the outcomes of the journal’s 1970s internationalization efforts. Notably, 50 of these pivotal publications were produced in the years 1972 and 1974 alone (Fig. 5), underscoring the beneficial impact of publishing in English on citation numbers—a key factor for enduring relevance in the global scientific community (Starke 1998; Francisco 2015; Gzoyan et al. 2023).

The analysis reveals a linguistic shift in the composition of the most-cited list, transitioning from a predominance of German-language articles before the late 1960s to a decisive majority of English-language articles post-1970. By 1971, English-language publications constituted over half of the top 100 cited works (Fig. 6), reflecting the journal’s successful adaptation to the international scientific publishing landscape (Starke 1998).

Among these highly cited articles, research on the cholinergic and adrenergic systems is particularly prominent, representing 21% of the list, followed by studies on pharmacodynamics and the dopaminergic system (Fig. 7; Fig. S2). This thematic emphasis is in part due to the foundational discoveries by Otto Loewi and Sir Henry Dale on neurotransmission, which have sparked extensive research into these biological systems (Tansey 2006; McCoy and Tan 2014). Technological advancements in the 1950s that improved acetylcholine detection also played a critical role, facilitating expanded research that coincided with heightened interest due to the military application of nerve agents during and post-World War II (Dacre 1984; Warburton and Wesnes 1985; John et al. 2018; Amend et al. 2020; Hrvat and Kovarik 2020).

Interest in the cholinergic and adrenergic systems surged until the 1990s before Naunyn–Schmiedeberg’s Archives of Pharmacology moved towards immunopharmacology and drugs for the treatment of malignant tumor diseases in the new millennium (Hornykiewicz 1966; Dats et al. 2023). The journal’s 1970s internationalization initiatives played a crucial role in enhancing citation metrics and expanding its international stature (Starke 1998; Francisco 2015; Hattori and Seifert 2023). Earlier periods also witnessed citation surges, notably in 1963 and 1968, thanks to seminal works by Huković and Muscholl and the influential article by Thoenen and Tranzer (1968) on dopaminergic neurons, which is the period’s most cited work with 717 citations (Table 1, last accessed 02.01.2024). Importantly, alongside the journal’s significant contributions to neuroscience, this study highlights a pivotal moment in Parkinson’s disease research. Consequently, the mid-1960s are marked by an improvement in Parkinson’s therapy, significantly contributing to the scientific understanding of dopamine’s function. The Thoenen and Tranzer article specifically explored the effects of 6-hydroxydopamine (6-OHDA) on dopaminergic neurons, demonstrating its potential to induce Parkinson’s syndrome in experimental models. This critical insight into 6-OHDA has since been instrumental in the study and treatment of Parkinson’s disease, further emphasizing the journal’s enduring influence on neurological research and therapy (Hornykiewicz 1966; Thoenen and Tranzer 1968; Simola et al. 2007; Fahn 2008; Li and Le 2017).

**Table 1:** Top 100 most-cited articles (Original Papers; last accessed 02.01.24); *en* English, *ger* German

Rank	Citations	Title	Topics	Language	Authors	Pub year	Volume	Issue	Pages	City	
1	717	Chemical sympathectomy by selective destruction of adrenergic nerve endings with 6-hydroxydopamine	Dopaminergic system	en	H. Thoenen and J. P. Tranzer	1968	261	3	pp. 271–288	Basel	
2	547	Simultaneous measurement of tyrosine and tryptophan hydroxylase activities in brain in Vivo using an inhibitor of the aromatic amino acid decarboxylase	Serotonergic system	en	A. Carlsson, J. N. Davis, W. Kehr, Margit Lindqvist, and C. V. Atack	1972	275	2	pp. 153–168	Gothenburg	
3	275	Comparison of the effects of clonidine on pre-and postsynaptic adrenoceptors in the rabbit pulmonary artery	Cholinergic and adrenergic system	en	K. Starke, H. Montel, W. Gayk, and R. Merker	1974	285	2	pp. 133–150	Essen	
4	258	A method for the determination of 3,4-dihydroxyphenylalanine (DOPA) in brain	Dopaminergic system	en	W. Kehr, A. Carlsson, and M. Lindqvist	1972	274	3	pp. 273–280	Gothenburg	
5	249	Alpha sympathomimetic inhibition of adrenergic and cholinergic transmission in the rabbit heart	Cholinergic and adrenergic system	en	K. Starke	1972	274	1	pp. 18–45	Essen	
6	216	The effect of diazepam on spinal cord activities: Possible sites and mechanisms of action	Antiepileptic and benzodiazepine drugs	en	P. Polc, H. Möhler, and W. Haefely	1974	284	4	pp. 319–337	Basel	
7	209	Die Noradrenalin-Abgabe aus dem isolierten Kaninchenherzen bei sympathischer Nervenreizung und ihre pharmakologische Beeinflussung	Cholinergic and adrenergic system	ger	S. Huković and E. Muscholl	1962	244	1	pp. 81–96	Mainz	
8	168	Concentration and origin of choline in the rat brain	Cholinergic and adrenergic system	en	K. Dross and H. Kewitz	1972	274	1	pp. 91–106	Berlin	
9	167	A muscarinic inhibition of the noradrenaline release evoked by postganglionic sympathetic nerve stimulation	Cholinergic and adrenergic system	en	K. Löffelholz and E. Muscholl	1969	265	1	pp. 1–15	Mainz	
10	164	Conjoint native and orthophthaldialdehyde-condensate assays for the fluorimetric determination of 5-hydroxyindoles in brain	Serotonergic system	en	Colin Atack and Margit Lindqvist	1973	279	3	pp. 267–284	Gothenburg	
11	163	Adenosine release from isolated fat cells and its significance for the effects of hormones on cyclic 3′,5′-AMP levels and lipolysis	Purinergic system	en	U. Schwabe, R. Ebert, and H. C. Erbler	1973	276	2	pp. 133–148	Hanover	
12	156	Biochemistry and pharmacology of the crotoxin complex	Toxicology	en	K. Rübsamen, H. Breithaupt, and B. Habermann	1971	270	3	pp. 274–288	Giessen	
13	152	Die Wirkung von Pharmaka auf die Elimination von Noradrenalin aus der Perfusionsflüssigkeit und die Noradrenalinaufnahme in das isolierte Herz	Pharmacokinetics	ger	R. Lindmar and E. Muscholl	1964	247	5	pp. 469–492	Mainz	
14	148	Action of peptides and other algesic agents on paravascular pain receptors of the isolated perfused rabbit ear	Pain pharmacology	en	H. Juan and F. Lembeck	1974	283	2	pp. 151–164	Graz	
15	145	Über das sympathicomimetische pressorische Prinzip des Harns („Urosympathin “)	Pharmacodynamics	en	Peter Holtz, Karl Credner, and Günther Kroneberg	1947	204	1	pp. 228–243	Rostock	
16	144	Zur Frage der zentralen Übertragung afferenter Impulse	Pharmacodynamics	ger	F. Lembeck	1953	219	3	pp. 119–120	Graz	
17	143	Die Wirkung von Diazepam auf die präsynaptische Hemmung und andere Rückenmarksreflexe	Antiepileptic and Benzodiazepine Drugs	ger	Robert F. Schmidt, Martin E. Vogel, and Manfred Zimmermann	1967	258	1	pp. 69–82	Heidelberg	
18	140	Influence of extracellular noradrenaline on the stimulation-evoked secretion of noradrenaline from sympathetic nerves: Evidence for an α-receptor-mediated feed-back inhibition of noradrenaline release	Cholinergic and Adrenergic System	en	K. Starke	1972	275	1	pp. 11–23	Essen	
19	132	Embryotoxic effects in mice treated with 2,4,5-trichlorophenoxyacetic acid and 2,3,7,8-tetrachlorodibenzo-p-dioxin	Toxicology	en	Diether Neubert and Imke Dillmann	1972	272	3	pp. 243–264	Berlin	
20	131	Measurement of hydrophobicity, surface activity, local anaesthesia, and myocardial conduction velocity as quantitative parameters of the non-specific membrane affinity of nine β-adrenergic blocking agents	Local anesthetics and inhalation/injection anesthetics	en	D. Hellenbrecht, B. Lemmer, G. Wiethold, and H. Grobecker	1973	277	2	pp. 211–226	Frankfurt a. M	
21	129	Effect of a transverse cerebral hemisection on 5-hydroxytryptamine metabolism in the rat brain	Serotonergic system	en	P. Bédard (Fellow of the Medical Research Council of Canada), A. Carlsson, and M. Lindqvist	1972	272	1	pp. 1–15	Gothenburg	
22	128	Effect of chronic transection on dopamine, noradrenaline and 5-hydroxytryptamine in the rat spinal cord	Dopaminergic system	en	Tor Magnusson	1973	278	1	pp. 13–22	Gothenburg	
23	127	Studies on the mechanism of action of dantrolene sodium	Pharmacodynamics	en	K. O. Ellis and J. F. Carpenter	1972	275	1	pp. 83–94	New York	
24	126	Pharmacological properties of cardiotoxin isolated from Formosan cobra venom	Toxicology	en	C. Y. Lee, C. C. Chang, T. H. Chiu, P. J. S. Chiu, T. C. Tseng, and S. Y. Lee	1968	259	4	pp. 360–374	Taipei	
25	122	Assay of phosphodiesterase with radioactively labeled cyclic 3′,5′-AMP as substrate	Purinergic system	en	G. Pöch	1971	268	3	pp. 272–299	Graz	
26	120	Experimental hypertension of the rat: Reciprocal changes of norepinephrine turnover in heart and brain-stem	Antihypertensive agents	en	K. Nakamura, M. Gerold, and H. Thoenen	1971	268	2	pp. 125–139	Basel	
27	119	Zur resorptionshemmenden Wirkung von Gallensäuren	Drugs for the treatment of disorders of lipid metabolism	ger	W. Forth, W. Rummel, and H. Glasner	1966	254	4	pp. 382–383	Homburg	
28	115	125I-labeled neurotoxin from clostridium botulinum A: Preparation, binding to synaptosomes and ascent to the spinal cord	Pharmacokinetics	en	E. Habermann	1974	281	1	pp. 47–56	Giessen	
29	114	Influence of morphine and naloxone on the release of noradrenaline from rat brain cortex slices	Pain pharmacology	en	H. Montel, K. Starke, F. Weber	1974	283	4	pp. 357–369	Essen	
30	113	Increase in brain dopamine after axotomy or treatment with gammahydroxybutyric acid due to elimination of the nerve impulse flow	Pharmacokinetics	en	Günter Stock, Tor Magnusson, Nils-Erik Andén	1973	278	4	pp. 347–361	Gothenburg	
31	111	Über den Wirkungsmechanismus einer neuen antihypertensiven Substanz mit Imidazolinstruktur	Antihypertensive agents	ger	W. Kobinger	1967	258	1	pp. 48–58	Vienna	
32	111	Activation of the central pathway of the baroreceptor reflex, a possible mechanism of the hypotensive action of clonidine	Antihypertensive agents	en	G. Haeusler	1973	278	3	pp. 231–246	Basel	
33	109	Positive inotropic effects of phenylephrine in the isolated rabbit papillary muscle mediated both by α-and β-adrenoceptors	Cholinergic and adrenergic system	en	H. J. Schümann, M. Endoh, and J. Wagner	1974	284	2	pp. 133–148	Essen	
34	103	Evidence that punctate intracerebral administration of 6-hydroxydopamine fails to produce selective neuronal degeneration	Dopaminergic system	en	Larry L. Butcher, Sheila M. Eastgate, and Gordon K. Hodge	1974	285	1	pp. 31–70	California	
35	99	Elektrophysiologische Messungen zur Strophanthinwirkung am Herzmuskel	Antiarrhythmic drugs	ger	J. Dudel and W. Trautwein	1957	232	2	pp. 393–407	Heidelberg	
36	99	Alpha-receptor-mediated modulation of transmitter release from central noradrenergic neurones	Cholinergic and adrenergic system	en	K. Starke and H. Montel	1973	279	1	pp. 53–60	Essen	
37	98	Effects on self-stimulation behavior of drugs influencing dopaminergic neurotransmission mechanisms	Dopaminergic system	en	Jeffrey M. Liebman and Larry L. Butcher	1973	277	3	pp. 305–318	California	
38	98	Der beschleunigte Abbau von Pharmaka in den Lebermikrosomen unter dem Einfluß von Luminal	Pharmacokinetics	ger	H. Remmer	1959	235	4	pp. 279–290	Berlin	
39	96	Potentiation of the effects of isoprenaline and noradrenaline by hydrocortisone in cat heart muscle	Cholinergic and adrenergic system	en	Alberto J. Kaumann	1972	273	1	pp. 134–153	Buenos Aires	
40	94	Synthesesteigerung und Abbauhemmung bei der Vermehrung der mikrosomalen Cytochrome P-450 und b-5 durch Phenobarbital	Pharmacokinetics	ger	Helmut Greim	1970	266	3	pp. 261–275	Tübingen	
41	93	Eine Methode zur direkten Messung der O-Demethylierung in Lebermikrosomen und ihre Anwendung auf die Mikrosomenhemmwirkung von SKF 525-A	Pharmacodynamics	ger	K. J. Netter	1960	238	2	pp. 292–300	Hamburg	
42	90	Suppression by dopamine-agonists of the ethanol-induced stimulation of locomotor activity and brain dopamine synthesis	Cholinergic and adrenergic system	en	Arvid Carlsson, Jörgen Engel, Ulf Strömbom, Torgny H. Svensson, and Bertil Waldeck	1974	283	2	pp. 117–128	Gothenburg	
43	88	Metabolic activation of halothane and its covalent binding to liver endoplasmic proteins in vitro	Local anesthetics and inhalation/injection anesthetics	en	H. Uehleke, K. H. Hellmer, and S. Tabarelli-Poplawski	1973	279	1	pp. 39–52	Tübingen	
44	87	Electrical events in cardiac adrenergic nerves and noradrenaline release from the heart induced by acetylcholine and KCl	Cholinergic and adrenergic system	en	G. Haeusler, H. Thoenen, W. Haefely, and A. Huerlimann	1968	261	5	pp. 389–411	Basel	
45	86	Neurally mediated control of enzymes involved in the synthesis of norepinephrine; are they regulated as an operational unit?	Cholinergic and adrenergic system	en	H. Thoenen, R. Kettlee, W. Burkard, and A. Saner	1971	270	2	pp. 146–160	Basel	
46	85	Effect of duration and frequency of stimulation on the presynaptic inhibition by α-adrenoceptor stimulation of the adrenergic transmission	Cholinergic and adrenergic system	en	E. S. Vizi, G. T. Somogyi, P. Hadházy, and J. Knoll	1973	280	1	pp. 79–91	Budapest	
47	83	Comparative involvement of dopamine and noradrenaline in rate-free self-stimulation in substantia nigra, lateral hypothalamus, and mesencephalic central gray	Dopaminergic system	en	Jeffrey M. Liebman and Larry L. Butcher	1974	284	2	pp. 167–194	California	
48	82	Effects of monocular deprivation in kittens	Pharmacodynamics	en	David H. Hubel and Torsten N. Wiesel	1964	248	6	pp. 492–497	Boston	
49	81	Mastzelldegranulierendes Peptid (MCD-Peptid) aus Bienengift: Isolierung, biochemische und pharmakologische Eigenschaften	Toxicology	ger	H. Breithaupt and E. Habermann	1968	261	3	pp. 252–270	Giessen	
50	80	The inhibition of the sarcoplasmic calcium pump by prenylamine, reserpine, chlorpromazine and imipramine	Cholinergic and adrenergic system	en	H. Balzer, M. Makinose, and W. Hasselbach	1968	260	5	pp. 444–455		Heidelberg
51	80	Der Einfluß von Thalidomid auf die Fertilität von Ratten im Generationsversuch über zwei Generationen	Immunopharmacology	ger	Rudolf Kopf, Dietrich Lorenz, and Ekkehard Salewski	1964	247	2	pp. 360–361		Cologne
52	80	Benzo[a]chinolizine, eine neue Körperklasse mit Wirkung auf den 5-Hydroxytryptamin- und Noradrenalin-Stoffwechsel des Gehirns	Serotonergic system	ger	A. Pletscher, H. Besendorf, and H. P. Bächtold	1958	232	3	pp. 499–506		Basel
53	79	Die Hemmung der Noradrenalin-Aufnahme des Herzens durch Reserpin und die Wirkung von Tyramin	Pharmacokinetics	ger	E. Muscholl	1960	240	3	pp. 234–241		Mainz
54	78	Characterization of the binding of benzodiazepines to human serum albumin	Antiepileptic and benzodiazepine drugs	en	W. Müller and U. Wollert	1973	280	3	pp. 229–237		Mainz
55	78	The influence of phenoxybenzamine and isopropylmethoxamine (BW 61–43) on some cardiovascular, metabolic, and histopathologic effects of norepinephrine infusions in dogs	Cholinergic and adrenergic system	en	H. M. Maling, M. A. Williams, B. Highman, J. Garbus, and J. Hunter	1964	248	1	pp. 54–72		Bethesda
56	78	Analysis of the compartments involved in the extraneuronal storage and metabolism of isoprenaline in the perfused heart	Cholinergic and adrenergic system	en	H. Bönisch, W. Uhlig, and U. Trendelenburg	1974	283	3	pp. 223–244		Würzburg
57	77	The mechanism of halothane binding to microsomal cytochrome P450	Local anesthetics and inhalation/injection anesthetics	en	D. Mansuy, W. Nastainczyk, and V. Ullrich	1974	285	4	pp. 315–324		Homburg
58	75	The biosynthesis of octopamine	Cholinergic and adrenergic system	en	K. Brandau (Visiting Scientist from Farbenfabriken Bayer AG) and J. Axelrod	1972	273	1	pp. 123–133		Bethesda
59	74	On the mechanism of the adrenergic nerve blocking action of bretylium	Cholinergic and adrenergic system	en	G. Haeusler, W. Haefely, and A. Huerlimann	1969	265	3	pp. 260–277		Basel
60	73	Superfusion of the hypothalamus with gamma-aminobutyric acid	Toxicology	en	A. Philippu, H. Przuntek, and W. Roensberg	1973	276	2	pp. 103–118		Würzburg
61	72	Der Einfluß verschiedener Ernährungszustände auf die Glykogenbildung im überlebenden Zwerchfell- und Fettgewebe der Ratte	Pharmacodynamics	ger	Franz X. Hausberger and Orest Jachtorowycz	1949	207	1	pp. 71–81		Erlangen
62	71	Vascular reactivity to 5-hydroxytryptamine and hypertension in the rat	Serotonergic system	en	G. Haeusler and L. Finch	1972	272	1	pp. 101–116		Basel
63	70	Cardiac glycosides: Correlations among Na + , K + -ATPase, sodium pump and contractility in the guinea pig heart	Drugs for treatment of heart failure and coronary heart disease	en	D. Ku, T. Akera, C. L. Pew, and T. M. Brody	1974	285	2	pp. 185–200		East Lansing
64	70	Studies on the antilipolytic effect of adenosine and related compounds in isolated fat cells	NO-cGMP system	en	R. Ebert and U. Schwabe	1973	278	3	pp. 247–259		Hanover
65	69	Quabain: Temporal relationship between the inotropic effect and the in vitro binding to, and dissociation from, (Na +/K +)-activated ATPase	Drugs for treatment of heart failure and coronary heart disease	en	T. Akera, S. I. Baskin, T. Tobin, and T. M. Brody	1973	277	2	pp. 112–123		East Lansing
66	68	Extraneuronal removal, accumulation and O-methylation of isoprenaline in the perfused heart	Pharmacokinetics	en	H. Bönisch and U. Trendelenburg	1974	283	2	pp. 191–218		Würzburg
67	67	Hemmung der Serotoninbildung durch α-Methyl-Dopa	Dopaminergic system	ger	E. ermann, H. Balzer, and J. Knell	1958	234	3	pp. 194–205		Frankfurt a. M
68	67	Effects of morphine on kinetics of 14C-dopamine in rat striatal slices	Pain pharmacology	en	B. Celsen and K. Kuschinsky	1974	284	2	pp. 159–165		Göttingen
69	67	A comparison of the kinetic properties of phosphofructokinase from bacterial, plant and animal sources	Pharmacokinetics	en	Oliver H. Lowry and Janet V. Passonneau	1964	248	2	pp. 185–194		St. Louis
70	67	Calcium-mediated action potentials in mammalian myocardium	Pharmacokinetics	en	H. Tritthart, R. Volkmann, R. Weiss, and A. Fleckenstein	1973	280	3	pp. 239–252		Freiburg
71	66	Different effects of lipolytic hormones and phosphodiesterase inhibitors on cyclic 3′,5′-AMP levels in isolated fat cells	Drugs for the treatment of disorders of lipid metabolism	en	U. Schwabe and R. Ebert (Recipient of a Deutsche Forschungsgemeinschaft scholarship)	1972	274	3	pp. 287–298		Hanover
72	66	Über das Vorkommen der Hypophysenhinterlappenhormone im Zwischenhirn	Pharmacodynamics	ger	Walther Hild	1951	213	1	pp. 139–153		Kiel
73	66	Der Ort der N-Oxydation des Anilins im höheren Tier	Pharmacodynamics	ger	M. Kiese and Hartmut Uehleke	1961	242	2	pp. 117–129		Munich
74	66	Permeation morphinartig wirksamer Substanzen an den Ort der antinociceptiven Wirkung im Gehirn in AbhÄngigkeit von ihrer Lipoidlöslichkeit nach intravenöser und nach intraventrikulÄrer Applikation	Pain pharmacology	ger	B. von Cube, Hj. Teschemacher, A. Herz, and R. Hess	1970	265	5	pp. 455–473		Munich
75	64	Central nervous α-adrenergic receptors and the mode of action of α-methyldopa	Dopaminergic system	en	A. Heise and G. Kroneberg	1973	279	3	pp. 285–300		Wuppertal-Elberfeld
76	64	The preparation of microsomes	Pharmacodynamics	en	Rosemarie von Jagow, Hermann Kampffmeyer, and Manfred Kinese	1965	251	1	pp. 73–87		Munich
77	64	Pre- and postjunctional supersensitivity of the mesenteric artery preparation from normotensive and hypertensive rats	Pharmacodynamics	en	G. Haeusler and W. Haefely	1970	266	1	pp. 18–33		Basel
78	63	Postmortal accumulation of 3-methoxytyramine in brain	Dopaminergic system	en	A. Carlsson, M. Lindqvist, and W. Kehr	1974	284	4	pp. 365–372		Gothenburg
79	63	Substanz P und Speichelsekretion	Substanz P	ger	F. Lembeck and K. Starke	1968	259	5	pp. 375–385		Tübingen
80	63	Synergism between phospholipase A and various peptides and SH-reagents in causing haemolysis	Toxicology	en	W. Vogt, P. Patzer, Lotte Lege, H. -D. Oldigs, and Gabriele Wille	1970	265	5	pp. 442–454		Göttingen
81	63	Further studies on the stimulation of hepatic microsomal drug metabolizing enzymes by DDT and its analogs	Toxicology	en	Larry G. Hart and James R. Fouts	1965	249	6	pp. 486–500		Iowa City
82	62	Functional and biochemical effects of d- and l-amphetamine on central catecholamine neurons	Dopaminergic system	en	Torgny H. Svensson	1971	271	2	pp. 170–180		Gothenburg
83	62	Pharmakologie des Tetrahydropapaverolins und seine Entstehung aus Dopamin	Dopaminergic system	ger	P. Holtz, K. Stock, and E. ermann	1964	248	5	pp. 387–405		Frankfurt a. M
84	62	Die Wirkung von Cocain, Guanethidin, Reserpin, Hexamethonium, Tetracain und Psicain auf die Noradrenalin-Freisetzung aus dem Herzen	Local anesthetics and inhalation/injection anesthetics	ger	Ruth Lindmar and E. Muscholl	1961	242	3	pp. 214–227		Mainz
85	62	Untersuchungen zum Wirkungsmechanismus des Phalloidins	Purinergic system	ger	M. Frimmer, J. Gries, and D. Hegner	1967	258	2	pp. 197–214		Giessen
86	62	Anorexigene Phenylalkylamine und Serotoninstoffwechsel	Serotonergic system	ger	Klaus Opitz	1967	259	1	pp. 56–65		Münster
87	62	Untersuchungen zum Mechanismus der Freisetzung von Brenzcatechinaminen durch Tyramin	Serotonergic system	ger	H. J. Schümann and A. Philippu	1961	241	2	pp. 273–280		Frankfurt a. M
88	61	Effects of drugs influencing monoamine mechanisms on the increase in brain dopamine produced by axotomy or treatment with gammahydroxybutyric acid	Dopaminergic System	en	Nils-Erik Andén, Tor Magnusson, and Günter Stock	1973	278	4	pp. 363–372		Gothenburg
89	61	Pharmacology of pyramidal tract cells in the cerebral cortex	Pharmacodynamics	en	T. W. Stone	1973	278	4	pp. 333–346		Aberdeen
90	61	Selective stimulation of the parasympathetic preganglionic nerve fibres in the excised and blood-perfused SA node preparation of the dog	Pharmacology of the kidney	en	Katsumi Kubota and Koroku Hashimoto	1973	278	2	pp. 135–150		Tohoku
91	60	Release of prostaglandins, a prostaglandin metabolite, slow-reacting substance and histamine from anaphylactic lungs, and its modification by catecholamines	Histaminergic system	en	R. Liebig, W. Bernauer, and B. A. Peskar	1974	284	3	pp. 279–293		Freiburg
92	60	Reduced pressor responses to stimulation of the locus coeruleus after lesion of the posterior hypothalamus	Pharmacodynamics	en	H. Przuntek and A. Philippu	1973	276	2	pp. 119–122		Würzburg
93	59	Über den Mechanismus der Catecholamin-Speicherung in den „chromaffinen Granula “ des Nebennierenmarks	Cholinergic and adrenergic system	ger	G. Taugner and W. Hasselbach	1966	255	3	pp. 266–286		Heidelberg
94	59	The effects of 5-hydroxytryptamine and some related compounds on the cat superior cervical ganglion in situ	Serotonergic system	en	W. Haefely	1974	281	2	pp. 145–165		Basel
95	58	The adrenal dopamine as an indicator of adrenomedullary hormone biosynthesis	Dopaminergic system	en	S. R. Snider and A. Carlsson	1972	275	4	pp. 347–357		Gothenburg
96	58	Biochemische Grundlagen der Diazoxid-Hyperglykämie	Drugs for treatment of heart failure and coronary heart disease	ger	G. Schultz, G. Senft, W. Losert, and R. Sitt	1966	253	3	pp. 372–387		Berlin
97	58	Größe und DNS-Synthese der Leber unter dem Einfluß körperfremder Stoffe	Pharmacodynamics	ger	I. Schlicht, W. Koransky, S. Magour, and R. Schulte-Hermann	1968	261	1	pp. 26–41		Berlin
98	58	Enzymatische Inaktivierung von Isonicotinsäurehydrazid im menschlichen und tierischen Organismus	Pharmacokinetics	ger	Rudolf Bönicke and Waltraud Reif	1953	220	4	pp. 321–333		Borstel
99	58	Über die Wirkung von Metyrapon auf den mikrosomalen Arzneimittelabbau	Pharmacokinetics	ger	K. J. Netter	1967	259	1	pp. 1–16		Mainz
100	58	Der Einfluß der Durchblutung auf die Resorption von Arzneimitteln aus dem Jejunum der Ratte	Pharmacokinetics	ger	H. Ochsenfahrt and D. Winne	1969	264	1	pp. 55–75		Tübingen

### Authors

Between 1947 and 1974, Naunyn–Schmiedeberg’s Archives of Pharmacology saw contributions from 2065 first authors. Detailed analysis including first, second, and last authorships identified those who published (Original Papers) most frequently within this timeframe. Leading the count was Manfred Kiese (1910–1983) with 47 publications, followed closely by Gerhard Zetler (1921–2007) and Gustav Kuschinsky (1904–1992), each with 29 publications. Ernst Habermann (1926–2001) and Peter Holtz (1902–1970) also made significant contributions with 27 and 26 publications, respectively (Fig. 9; Tab. S2).

Manfred Kiese, who completed his doctorate under Wolfgang Heubner (1877–1957) in 1935, showed remarkable productivity, especially between 1947 and 1949, by publishing 17 articles while leading the pharmacology laboratory at the University Hospital in Kiel, a position he assumed in 1947. Kiese’s research predominantly focused on the pharmacodynamics and kinetics of methemoglobin and hemoglobin, contributing 33 articles on these topics over the years (Philippu 2004–2021). Importantly, Kiese demonstrated early international engagement through his publications in English as early as 1963. One of his publications achieved remarkable recognition and is listed among the 100 most cited papers in the journal, in the 73rd place (Table 1).

Gerhard Zetler began publishing consistently in 1951, maintaining an average of two publications per year until 1974. Starting his career at Christian-Albrecht University in Kiel in 1949, Zetler moved to the Institute for Experimental and Clinical Pharmacology and Toxicology at the University Medical Center Schleswig–Holstein in Lübeck in 1964, serving as its first director (Philippu 2004–2021). His work predominantly explored substance P and resulted in a total of 18 publications in Kiel and 11 in Lübeck.

Gustav Kuschinsky, another prominent contributor with 29 articles, started publishing in the journal in 1947 and continued until 1968. After beginning his career under Paul Trendelenburg in Berlin, he moved to Tung Chi University in Shanghai in 1934 and later became a full professor at the German University in Prague in 1939 (Philippu 2004–2021). His research, which did not include any English publications, focused on the cholinergic and adrenergic systems, with a significant portion of his work being published during his tenure at the Johannes Gutenberg University in Mainz.

Ernst Habermann’s contributions, spanning from 1954 to 1974, were primarily in toxicology, including groundbreaking studies on bee venom (Apis mellifera). His work spanned two institutions: from 1954 to 1966 at the University of Würzburg and from 1966 to 1974 at the Justus Liebig University Giessen, with a transition to English publications starting in 1971 (Philippu 2004–2021). Two of his works were cited particularly often, which underlines their importance in the scientific community. These works are ranked 28th and 49th of the 100 most cited publications (Table 1).

Peter Holtz, with 26 articles, focused on the cholinergic and adrenergic system, particularly on noradrenaline, earning him the National Prize of the GDR. His work at the University of Rostock and later at Goethe University in Frankfurt am Main contributed significantly to the field. Two of his papers are among the 100 most cited articles in the journal, which also indicates that his work is highly recognized. These are ranked 15th and 62nd in the list of most cited papers (Table 1).

### Continent, countries, and cities

The geographical distribution of the publications (Original Papers) further highlights the journal’s initial European, particularly German, orientation, with 94% of articles coming from Europe until 1974 (Fig. S3). This dominance underscores the limited global reach of publications in the German language, which rarely gained significance beyond Europe (Bajerski 2011). North America’s 4% contribution is primarily attributed to works dedicated to Otto Krayer and Otto Loewi, indicating a Western-centric publication trend (Starke 1998). The concentration of publications in Germany, especially in the immediate post-war era, can be seen as an effort to mitigate the country’s international isolation and foster reintegration into the global scientific community. This period also saw a shift in focus from national prestige to the pursuit of international recognition among German scientists after the second world war (Ahlers et al. 2023).

A comparison of the present data with the results of Dats et al. (2023) for the period 1990 to 2020 reveals an increasing internationalization of the journal until 2020. Particularly, a significant increase in the representation of Asian publications can be observed. While the Asian continent was clearly underrepresented until 1974 with only 47 publications (Fig. S4), Asia positioned itself as the second most represented continent from 1990 to 2020. The analysis by Dats et al. (2023) also reveals an increase in publications from South America, with the number of publications increasing from an under-representation of only 20 publications up to 1974 to around 250 in the period from 1990 to 2020, with the majority of these publications coming from Brazil. In contrast, the number of publications from Africa and Australia did not change relatively in the observed period by Dats et al. (2023).

In the analyzed period from 1947 to 1974, an overwhelming 84% of publications in Naunyn–Schmiedeberg’s Archives of Pharmacology originated from Germany, with Austria and Switzerland contributing 5% and 4%, respectively (see Fig. S4). These data underscore that during this period, 93% of the journal's publications were from German-speaking countries, aligning with the perception of the journal as predominantly German in its focus (Fig. 3). Post-World War II, a modest 10% of contributions came from non-German-speaking countries. However, the 1970s marked a pivotal shift towards internationalization, leading to a significant increase in contributions from outside the German-speaking countries. By the late 1990s, international contributions constituted 60% of the total publications, reflecting the journal’s successful global integration (Starke 1998; Hamel 2007).

**Fig. 10:**
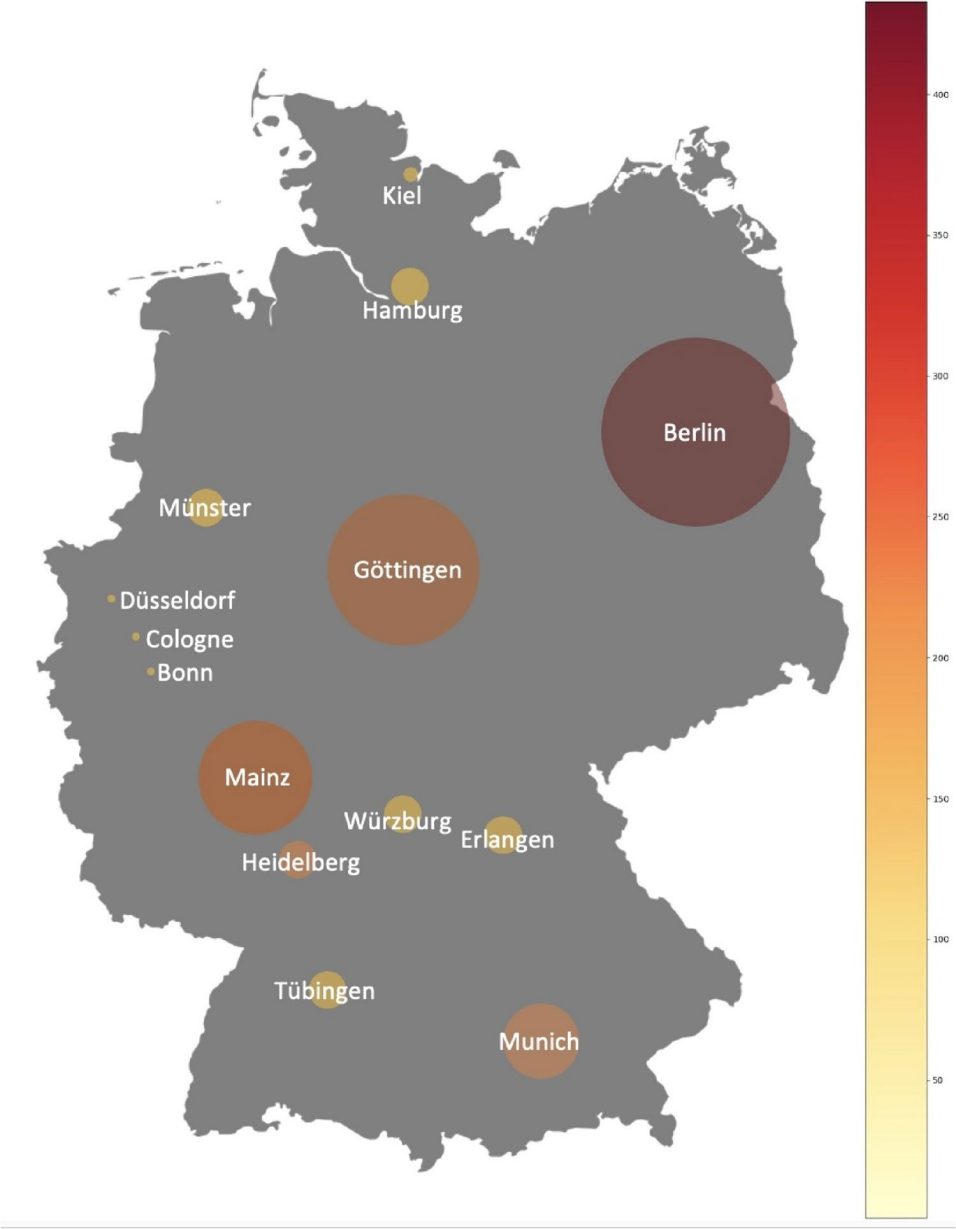
Heatmap of German cities; hotspot of publication (Original Papers) cities (1947–1974)

**Fig. 11:**
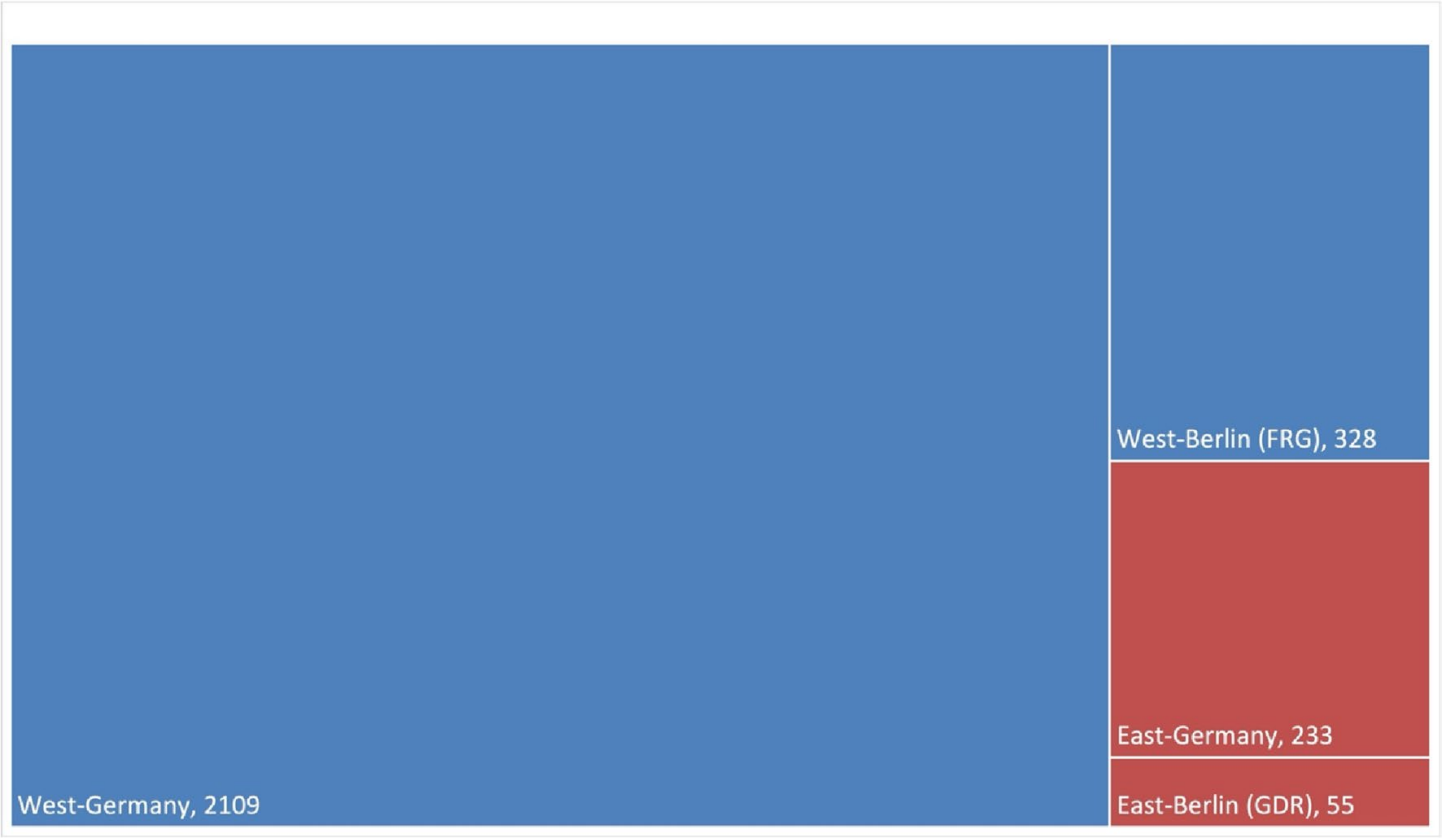
Geographical distribution of publications (Original Papers): West-Germany and East-Germany including Berlin (1947–1974). Formally, West-Berlin did not belong to the FRG, but the political system was similar to that of West-Germany (FRG)

**Fig. 12:**
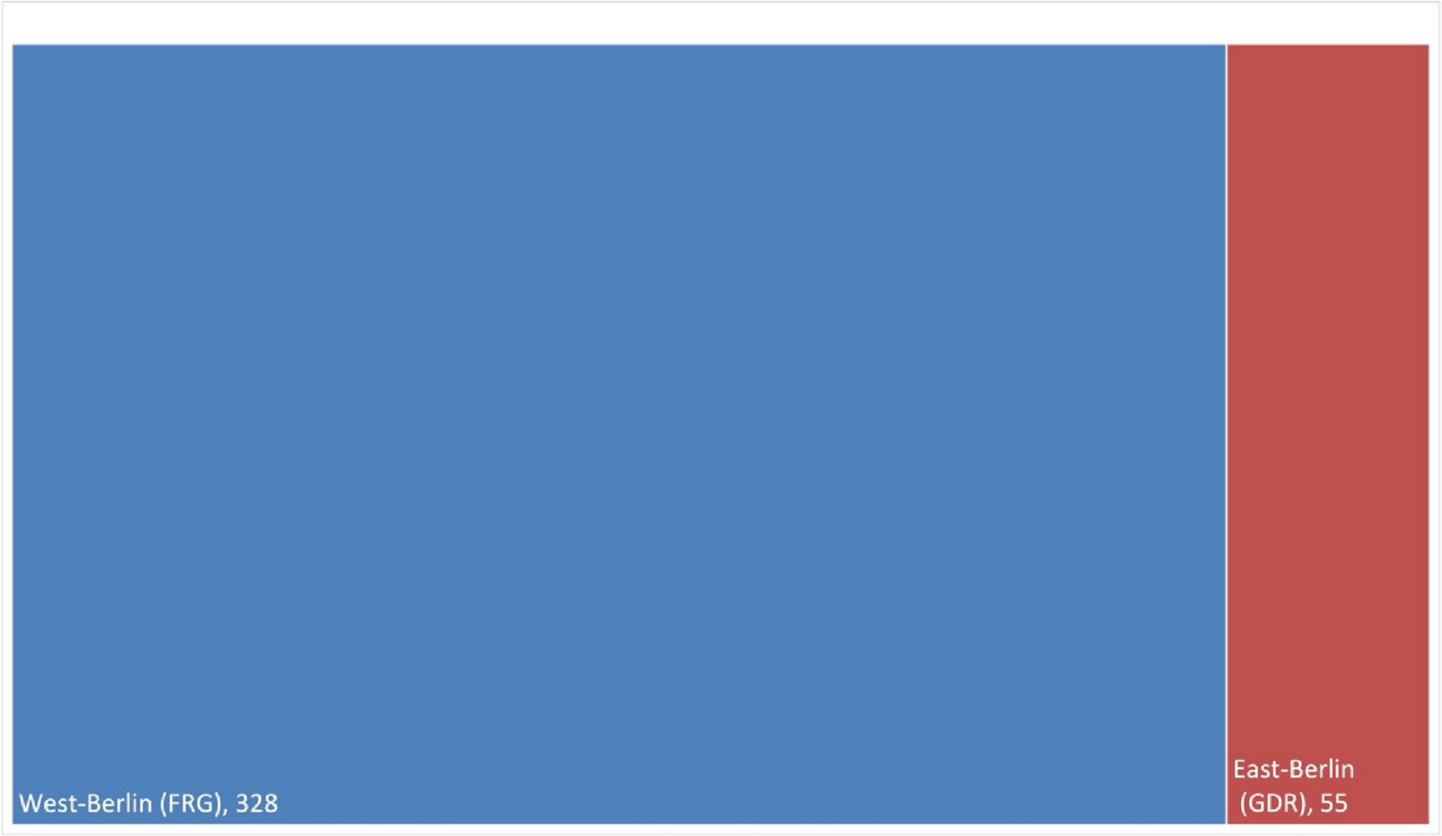
Publication distribution (Original Papers) between East-Berlin and West-Berlin (1947–1974). Formally, West-Berlin did not belong to the FRG, but the political system was similar to that of West-Germany (FRG)

Figures 10 and 11 show that most publications (Original Papers) in the period from 1947 to 1974, with a total of 1971 publications, came from West-Germany. Whereas 616 publications came from East-Germany, a closer look shows that 328 of these works were published in West-Berlin. Taking this categorization into consideration, only about 12% of the publications, namely 288, came from the geographical area of the former GDR; 55 of these works were published in East-Berlin. This publication distribution across German cities indicates a pronounced clustering in Western Germany (Figs. 10, 11, and 12).

**Fig. 13:**
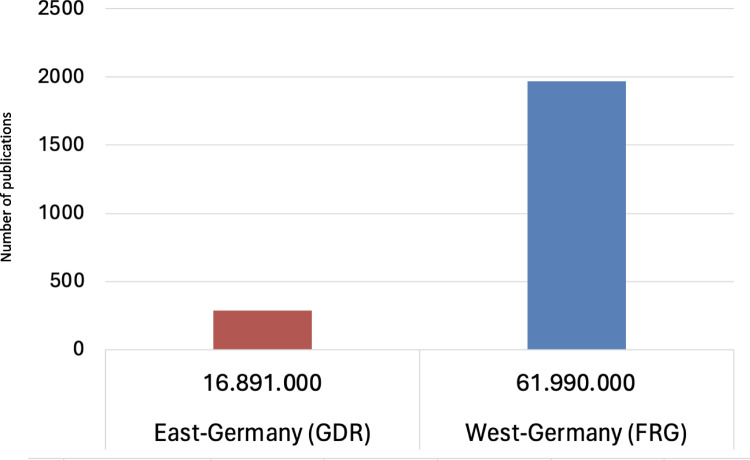
Total publications (Original Papers) and population figures in 1974 for East-Germany (GDR) and West-Germany (FRG) (Statistisches Bundesamt Demografische Aspekte Deutschland n.d.)

In 1974, the population of the GDR was 16.891 millions and the FRG 61.99 millions (Statistisches Bundesamt Demografische Aspekte Deutschland n.d.). West-Germany produced 1971 publications (Original Papers) in total, whereas East-Germany produced 288 publications (Original Papers; Fig. 11). In terms of population size, the FRG, including West-Berlin, produced around 31.8 publications per million inhabitants, while the GDR, including East-Berlin, recorded around 17.1 publications per million inhabitants. Although the population in West-Germany is almost four times as high as in East Germany, East Germany was still quite productive with 17.1 publications (original paper) (Fig. 13 and Table 2). However, the discrepancy increases when Berlin is considered individually in East and West. West-Berlin had a population of around 2.1 million people (1974), while East Berlin had a population of around 1.1 million (1974) (Statistisches Bundesamt Demografische Aspekte Deutschland n.d.). During the time between 1947–1974, publications per 1 million inhabitants in West-Berlin amounted to 155, whereas publications per 1 million inhabitants in East-Berlin in 1974 amounted to 51 (Table 2). These figures illustrate the higher scientific productivity in the FRG compared to the GDR (Fig. 13). Nonetheless, the figures also show that even with limited financial resources, one can be scientifically productive.

**Table 2:** Comparative publication rates (Original Papers) per 1 million population in West-Germany and East-Germany and Berlin (separted into East-Berlin and West-Berlin) (1974) (Statistisches Bundesamt Demografische Aspekte Deutschland n.d.)

Region	Population 1974	Total publications (Original Papers)	Publications per 1 million population
West-Germany (FRG)	61,990,000	1971	31.79
East-Germany (GDR)	16,891,000	288	17.05
West-Berlin	2,115,311	328	155.07
East-Berlin	1,086,400	55	50.65

**Fig. 14:**
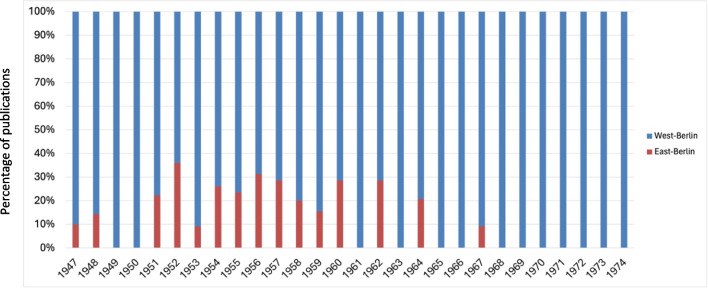
Comparative analysis of publication rates (Original Papers) in West-Berlin and East-Berlin between 1947 and 1974

Berlin emerged as the leading city in terms of publication volume (Original Papers) with 383, significantly outpacing Göttingen (rank 2) and Mainz (rank 3) (Fig. 10; Fig. S5 and S7). A closer look at Berlin allows us to recognize differences between the pharmacological institutes in West-Berlin and East-Berlin and they publication rates over time (Fig. 14). Between 1947 and 1974, the pharmacology institutes in West-Berlin were responsible for over 85% of publications (328 Original Papers), clearly surpassing the institutes in East-Berlin (55 Original Papers) (Fig. 12).

Excluding 1952, when East-Berlin institutes in the GDR contributed to approximately 36% of that year’s publications, West-Berlin institutes led in scientific output. Post-1952, East-Berlin’s publication numbers dwindled, halting almost entirely after 1962, with rare exceptions in 1964 and 1967 (Fig. 14). This decline is linked to the Cold War’s deepening, particularly after 1963, when East German pharmacologists were barred from participating in DGPT meetings. This restriction severely curtailed, if not entirely severed, scientific collaborations between pharmacologists from East and West Germany (Starke 1998).

In the aftermath of World War II, the DGPT showcased remarkable resilience and inclusivity, keeping its membership unified across the East–West divide. Early post-war gatherings, like the significant 1948 Düsseldorf meeting, saw participation from both East- and West-German pharmacologists. However, the intensifying Cold War tensions and Soviet policies made it impossible for East German members to participate, prompting them to form a separate society. Despite these challenges, the DGPT remained committed to fostering scientific dialogue and cooperation across the geopolitical divide, until external pressures necessitated a separate organization for East-German pharmacologists (Starke 1998).

Between 1951 and 1967, the presence of East-German pharmacologists in the Naunyn–Schmiedeberg’s Archives of Pharmacology significantly diminished, with a notable focus on research on drugs of treatment of heart failure and coronary heart disease, evidenced by 22 out of 49 original papers (Fig. 14). This focus mirrors the rise in cardiovascular disease mortality rates noted in the Soviet Union, a trend that the GDR likely experienced due to comparable socio-economic and environmental factors (Cooper 1983; Jargin 2017). With the intensification of the Cold War and the consequent isolation, East-German researchers shifted their contributions to “Die Pharmazie,” a journal established in 1946 in the GDR under the challenging conditions of the Soviet Occupation Zone. Remarkably, despite initial contributions from both Eastern and Western authors, political tensions also led to a decline in cross-border collaborations, particularly after the erection of the Berlin Wall. Nonetheless, “Die Pharmazie” continued to prioritize scientific inquiry over political content, gradually increasing its English-language publications and maintaining its significance as a scientific platform originating from the GDR (Friedrich and Helmstädter 2020).

Shortly after World War II, Germany was divided into four zones controlled by the Allies, leading to the formation of two separate states in 1949. The Federal Republic of Germany (FRG), formed from the western zones occupied by the United States, the United Kingdom, and France, adopted a capitalist system and embraced western democratic values, rapidly becoming integrated into the western bloc. Established in the Soviet-occupied zone, the German Democratic Republic (GDR) adopted a socialist economy and centralized governance and aligned itself with the communist ideology of the Soviet Union. This partition embodied the ideological fissure of the Cold War, with the FRG and the GDR each serving as outpost states for the competing Western and Eastern blocs (Kalberg 1991; Kastner 2002; Berger 2003).

West-Berlin’s prominence is attributed to the city’s vigorous push towards internationalization post-war and substantial financial support from the American occupation zone aimed at revitalizing academic activities. This strategic and financial backing significantly contributed to the resurgence of scientific work in West-Berlin, positioning it as a central hub for pharmacological research and publication (Philippu 2004–2021; Heinsohn and Nicolaysen 2021; Dats et al. 2023).

Dats et al. (2023) document a notable decline in Berlin’s output from 1990 to 2020 compared to the earlier period examined. Nonetheless, during this latter period, Berlin remains the epicenter for scholarly output in Eastern Germany. The enduring effects of post-World War II financial investments have had a long-term beneficial influence on academic institutions well into the millennium transition (Philippu 2004–2021; Heinsohn and Nicolaysen 2021; Dats et al. 2023).

Moreover, Freiburg ascended to the first position in the 1990–2020 timeframe, surpassing Berlin as the preeminent city. Conversely, Göttingen, previously ranked second, saw a significant reduction in its publication activity. Bonn, ranked twelfth up to 1974, advanced to second place during the 1990–2020 span (Dats et al. 2023; Fig. S7).

This analysis reveals dynamic shifts in publication rates (Original Papers) across various cities, with significant fluctuations observed. Despite these changes, the overarching pattern of publication concentration in Western Germany has persisted through many decades (Dats et al. 2023; Fig. 10). 

### Limitations and future studies

The bibliometric analysis conducted through data extraction from SpringerLink with Phython and Beautiful Soup provided a comprehensive yet focused insight into Naunyn–Schmiedeberg’s Archives of Pharmacology, centering on “Original Papers”. This approach, however, offered a limited view, excluding a variety of content such as short papers, DGPT spring meetings, abstracts, reviews, and reports etc. that also form an integral part of the journal’s content. The exclusion of reviews (SN-Type) was a strategic decision for this study, setting the stage for a subsequent study dedicated to exploring reviews in Naunyn–Schmiedeberg’s Archives of Pharmacology from its establishment in 1873 to the current day. The methodology described in this paper permits an in-depth evaluation over the journal’s full 150-year history. Investigating the initial resistance to English language adoption by Dr. Springer, the publisher, could also shed light on internal dynamics and resistance to internationalization within the journal’s editorial board, which saw the journal as distinctly German.

## Conclusions

The decline in publication numbers (Original Papers) in the early 1950s (Fig. 1) marked a critical juncture for Naunyn–Schmiedeberg’s Archives of Pharmacology, reflecting broader shifts within the scientific community and highlighting the journal’s struggle with post-war isolation due to its adherence to German. This period underscored the limitations of a national focus amidst a rapidly globalizing research landscape (Starke 1998; Francisco 2015; Gzoyan et al. 2023). Recognizing these challenges, the journal embarked on a path towards internationalization in the late 1960s, a move symbolized by the adoption of English for publications by 1973, which was pivotal in revitalizing the journal’s relevance and broadening its audience (Starke 1998; Dats et al. 2023; Hattori and Seifert 2023).

By the end of the 1990s, the composition of the journal’s authorship had dramatically shifted, transitioning from a predominantly German-centric outlet to one enriched by diverse international contributions (Dats et al. 2023). This transformation not only mirrors the evolving dynamics of scientific inquiry but also highlights the essential role of internationalization in fostering scientific collaboration and communication. The adoption of English as the lingua franca of science, while facilitating wider discourse, also introduces challenges for non-English speaking scientists, prompting reflections on access and equity within the global knowledge community (Tardy 2004; Di Bitetti and Ferreras 2017; O’Neil 2018).

This narrative extends beyond bibliometric analysis to engage with the discourses of internationality and scientific legitimacy, challenging the community to address the complexities of linguistic dominance. It underscores the necessity for journals like Naunyn–Schmiedeberg’s Archives of Pharmacology to navigate globalization thoughtfully, promoting scientific excellence and inclusivity (Phillipson 2009; Ahn et al. 2017; O’Neil 2018; Ahlers et al. 2023).

From its origins as a German journal to its current status as an internationally recognized publication, Naunyn–Schmiedeberg’s Archives of Pharmacology illustrates this interplay between tradition and innovation in scientific publishing. 

## Supplementary information

Below is the link to the electronic supplementary material.

Supplementary file1 (DOCX 456 KB)

## Data Availability

All source data for this study are available upon reasonable request from the authors.

## Abbreviations

DGPT: Deutsche Gesellschaft für experimentelle und klinische Pharmakologie und Toxikologie (German Society for Experimental and Clinical Pharmacology and Toxicology)
SN: Serial number (in context of publication types)
IF: Impact factor
DOI: Digital Object Identifier
CrossRef: An official DOI registration agency
Pandas: An open-source data analysis and manipulation tool in Python
Langdetect: Language detection library for Python
BeautifulSoup: A Python library for pulling data out of HTML and XML-files
GDR/DDR: German Democratic Republic (Deutsche Demokratische Republik)
FRG/BRD: Federal Republic of Germany (Bundesrepublik Deutschland)
FUB: Free University of Berlin (Freie Universität Berlin)
WWII: World War II

## References

[CR1] Ahlers AL, Hennings J, Schmidt F (2023) Internationalisierung im Fokus: Innenansichten aus dem deutschen Wissenschaftssystem. Monografie 41:4–30. https://www.diejungeakademie.de/media/pages/publikationen/internationalisierung-im-fokus/614e35ae01-1700660733/dja_debattenbeitrag_internationalisierung-im-fokus_a5_screen.pdf

[CR2] Ahn S, Chang CB, DeKeyser R, Lee-Ellis S (2017) Age effects in first language attrition: speech perception by Korean-English bilinguals. Lang Learn 67:694–733. 10.1111/lang.12252

[CR3] Amend N, Niessen KV, Seeger T et al (2020) Diagnostics and treatment of nerve agent poisoning—current status and future developments. Ann N Y Acad Sci 1479:13–28. 10.1111/nyas.14336 32198755 10.1111/nyas.14336

[CR4] Bajerski A (2011) The role of French, German and Spanish journals in scientific communication in international geography: French, German and Spanish journals in scientific communication. Area 43:305–313. 10.1111/j.1475-4762.2010.00989.x

[CR5] Berger S (2003) Former GDR historians in the reunified Germany: an alternative historical culture and its attempts to come to terms with the GDR past. J Contemp Hist 38:63–83. 10.1177/0022009403038001964

[CR6] Bettendorf G (1995) Loewi, Otto. In: Bettendorf G (ed) Zur Geschichte der Endokrinologie und Reproduktionsmedizin. Springer, Berlin Heidelberg, Berlin, Heidelberg, pp 357–358

[CR7] Cooper RS (1983) Epidemiologic features of recent trends in coronary heart disease in the Soviet Union. J Am Coll Cardiol 2:557–564. 10.1016/S0735-1097(83)80285-X 6875119 10.1016/s0735-1097(83)80285-x

[CR8] Dacre JC (1984) Toxicology of some anticholinesterases used as chemical warfare agents - a review. In: Brzin M, Barnard EA, Sket D (eds) Cholinesterases. De Gruyter, Berlin, Boston, pp 415–426

[CR9] Dats LB, Von Haugwitz F, Seifert R (2023) Bibliometric development of Naunyn–Schmiedeberg’s Archives of Pharmacology. Naunyn-Schmiedeberg’s Arch Pharmacol 396:43–61. 10.1007/s00210-022-02307-2 36280660 10.1007/s00210-022-02307-2 PMC9592544

[CR10] Davydova JG (2020) English in Germany: evidence from domains of use and attitudes. Russian Journal of Linguistics 24:687–702. 10.22363/2687-0088-2020-24-3-687-702

[CR11] Di Bitetti MS, Ferreras JA (2017) Publish (in English) or perish: the effect on citation rate of using languages other than English in scientific publications. Ambio 46:121–127. 10.1007/s13280-016-0820-7 27686730 10.1007/s13280-016-0820-7 PMC5226904

[CR12] Fahn S (2008) The history of dopamine and levodopa in the treatment of Parkinson’s disease: dopamine and levodopa in the treatment of PD. Mov Disord 23:S497–S508. 10.1002/mds.22028 18781671 10.1002/mds.22028

[CR13] Francisco JS (2015) Internationale Kooperationen: ein Schlüssel zu wissenschaftlichem Erfolg. Angew Chem 127:15196–15197. 10.1002/ange.201505267

[CR14] Friedrich C, Helmstädter A (2020) Seventy-five volumes of “Die Pharmazie” – a historical review. Pharmazie 75:289–293. 10.1691/ph.2020.0449 32635968 10.1691/ph.2020.0449

[CR15] Gzoyan E, Mirzoyan A, Sargsyan A et al (2023) International visibility of Armenian domestic journals: the role of scientific diaspora. Journal of Data and Information Science 8:93–117. 10.2478/jdis-2023-0011

[CR16] Hamel RE (2007) The dominance of English in the international scientific periodical literature and the future of language use in science. AILA 20:53–71. 10.1075/aila.20.06ham

[CR17] Hattori Y, Seifert R (2023) Reflections on the 150th anniversary of Naunyn–Schmiedeberg’s Archives of Pharmacology: past, challenges, and future. Naunyn-Schmiedeberg’s Arch Pharmacol 396:1–3. 10.1007/s00210-022-02321-4 10.1007/s00210-022-02321-4 PMC9788996 36336742

[CR18] Heinsohn K, Nicolaysen R (eds) (2021) Belastete Beziehungen: Studien zur Wirkung von Exil und Remigration auf die Wissenschaften in Deutschland nach 1945. Wallstein Verlag, Göttingen

[CR19] Herken H (1999) Die Berliner Pharmakologie in der Nachkriegszeit: Erinnerungen an ein Stück bewegter Universitätsgeschichte der Jahre 1945–1960. Springer, Berlin, New York

[CR20] Hornykiewicz O (1966) Dopamine (3-hydroxytyramine) and brain function. Pharmacol Rev 18:925–964 5328389

[CR21] Jargin SV (2017) Cardiovascular mortality in Russia: a comment. Cardiovasc Diagn Ther E13–E14, People’s Friendship University of Russia, Moscow, Russia, pp 60–84. 10.21037/cdt.2017.08.03 10.21037/cdt.2017.08.03 PMC5752834 29302472

[CR22] John H, Van Der Schans MJ, Koller M et al (2018) Fatal sarin poisoning in Syria 2013: forensic verification within an international laboratory network. Forensic Toxicol 36:61–71. 10.1007/s11419-017-0376-7 29367863 10.1007/s11419-017-0376-7 PMC5754388

[CR23] Kalberg S (1991) The hidden link between internal political culture and cross-national perceptions: divergent images of the Soviet Union in the United States and the Federal Republic of Germany. Theory Cult Soc 8:31–55. 10.1177/026327691008002002

[CR24] Kastner J (2002) The Berlin Crisis and the FRG, 1958–62. In: Gearson JPS, Schake K (eds) The Berlin Wall Crisis. Palgrave Macmillan UK, London, pp 125–146

[CR25] Koch-Weser J, Schechter PJ (1978) Schmiedeberg in Strassburg 1872–1918: The making of modern pharmacology. Life Sci 22:1361–1371. 10.1016/0024-3205(78)90099-1 351320 10.1016/0024-3205(78)90099-1

[CR26] Li S, Le W (2017) Milestones of Parkinson’s Disease Research: 200 years of history and beyond. Neurosci Bull 33:598–602. 10.1007/s12264-017-0178-2 28895075 10.1007/s12264-017-0178-2 PMC5636740

[CR27] Löffelholz K (2011) The persecution of pharmacologists in Nazi Germany and Austria. Naunyn-Schmied Arch Pharmacol 383:217–225. 10.1007/s00210-010-0560-3 10.1007/s00210-010-0560-3 20848274

[CR28] McCoy A, Tan S (2014) Otto Loewi (1873–1961): Dreamer and Nobel laureate. Singapore Med J (smedj) 55:3–4. 10.11622/smedj.2014002 10.11622/smedj.2014002 PMC4291908 24452970

[CR29] O’Neil D (2018) English as the lingua franca of international publishing. World Englishes 37:146–165. 10.1111/weng.12293

[CR30] Philippu A (2004–2021) Geschichte und Wirken der pharmakologischen, klinisch-pharmakologischen und toxikologischen Institute im deutschsprachigen Raum, vol I-VI. Berenkamp, Innsbruck, Austria

[CR31] Phillipson R (2009) Linguistic imperialism. Nachdr. Oxford Univ. Press, Oxford

[CR32] Python Software Foundation (2021) Python Software Foundation. In: Python.org. https://www.python.org. Accessed 25 Feb 2024

[CR33] Richardson L (2021) beautifulsoup4: Screen-scraping library. https://www.crummy.com/software/BeautifulSoup/. Accessed 25 Feb 2024

[CR34] Rubin RP (2014) The evolution of the discipline of pharmacology amid an era of global turbulence: the unique contributions of Otto Krayer (1899–1982). J Med Biogr 22:127–135. 10.1177/0967772014530798 24906402 10.1177/0967772014530798

[CR35] Seglen PO (1998) Citation rates and journal impact factors are not suitable for evaluation of research. Acta Orthop Scand 69:224–229. 10.3109/17453679809000920 9703393 10.3109/17453679809000920

[CR36] Seifert R (2018) Basiswissen Pharmakologie. Springer, Berlin Heidelberg, Berlin, Heidelberg

[CR37] Simola N, Morelli M, Carta AR (2007) The 6-Hydroxydopamine model of parkinson’s disease. Neurotox Res 11:151–167. 10.1007/BF03033565 17449457 10.1007/BF03033565

[CR38] Springer Link (2024) Naunyn-Schmiedeberg’s archives of pharmacology | Volumes and issues. In: SpringerLink. https://link.springer.com/journal/210/volumes-and-issues. Accessed 25 Feb 2024

[CR39] Starke K (1998) A History of Naunyn-Schmiedeberg’s Archives of Pharmacology: Naunyn-Schmiedeberg’s Arch Pharmacol 358:1–109. 10.1007/PL00005229 9721010 10.1007/pl00005229

[CR40] Statistisches Bundesamt Demografische Aspekte Deutschland (n.d.). In: Statistisches Bundesamt. https://www.destatis.de/DE/Themen/Querschnitt/Demografischer-Wandel/textbaustein-taser-blau-bevoelkerungszahl.html. Accessed 3 Mar 2024

[CR41] Tansey EM (2006) Henry Dale and the discovery of acetylcholine. CR Biol 329:419–425. 10.1016/j.crvi.2006.03.012 10.1016/j.crvi.2006.03.012 16731499

[CR42] Tardy C (2004) The role of English in scientific communication: lingua franca or Tyrannosaurus rex? J Engl Acad Purp 3:247–269. 10.1016/j.jeap.2003.10.001

[CR43] Thoenen H, Tranzer JP (1968) Chemical sympathectomy by selective destruction of adrenergic nerve endings with 6-hydroxydopamine. Naunyn-Schmiedebergs Arch Pharmak u Exp Path 261:271–288. 10.1007/BF00536990 10.1007/BF00536990 4387076

[CR44] Vinther S, Rosenberg J (2012) Impact factor trends for general medical journals: non-English-language journals are lacking behind. Swiss Med Wkly. 10.4414/smw.2012.13572 23015525 10.4414/smw.2012.13572

[CR45] Warburton DM, Wesnes K (1985) Historical overview of research on cholinergic systems and behavior. In: Singh MM, Warburton DM, Lal H (eds) Central Cholinergic Mechanisms and Adaptive Dysfunctions. Springer, US, Boston, MA, pp 1–35

[CR46] Weise-Pötschke S (2019) Entnazifizierung an der Akademie der Wissenschaft. Zeitschrift des Forschungsverbundes SED-Staat, 43 (2019), S 26–37. https://www.google.com/url?sa=t&source=web&rct=j&opi=89978449&url=https://zeitschrift-fsed.fu-berlin.de/index.php/zfsed/article/download/634/617&ved=2ahUKEwiPnciivLWFAxUbR_EDHeGAB-AQFnoECBIQAQ&usg=AOvVaw0s7ZZpMtdJtKUoxdKXCXk4

[CR47] Winkmann G, Schlutius S, Schweim HG (2002) Wie häufig werden deutschsprachige Medizinzeitschriften in der englischsprachigen Literatur zitiert? (Nachdruck) - Korreliert diese Rate mit dem Impact-Faktor, und wer zitiert? -. Klin Monatsbl Augenheilkd 219:72–78. 10.1055/s-2002-23505 11932815 10.1055/s-2002-23505

[CR48] Zehetbauer R, Von Haugwitz F, Seifert R (2022) Gender-specific analysis of the authors and the editorial board of Naunyn–Schmiedeberg’s Archives of Pharmacology from 2000 to 2020. Naunyn-Schmiedeberg’s Arch Pharmacol 395:39–50. 10.1007/s00210-021-02166-3 34622307 10.1007/s00210-021-02166-3 PMC8497184

