# Supplementary material for: Bibliometric analysis of Naunyn–Schmiedeberg’s Archives of Pharmacology (1947–1974)
Source: Naunyn Schmiedebergs Arch Pharmacol. 2024 Apr 23;397(9):7141–68. doi: 10.1007/s00210-024-03078-8 (PMC11422447; doi:10.1007/s00210-024-03078-8)
Supplement: Supplementary file 1 — Supplementary file1 (DOCX 456 KB) [file 210_2024_3078_MOESM1_ESM.docx]

**Supplemental Figures**

**Bibliometric Analysis of the Naunyn-Schmiedeberg’s Archives of Pharmacology (1947–1974)**

**Mert Erkan Basol and Roland Seifert**

**Institute of Pharmacology
Hannover Medical School
D-30625 Hannover, Germany
Corresponding author: Prof. Dr. Roland Seifert
seifert.roland@mh-hannover.de**

## Appendix


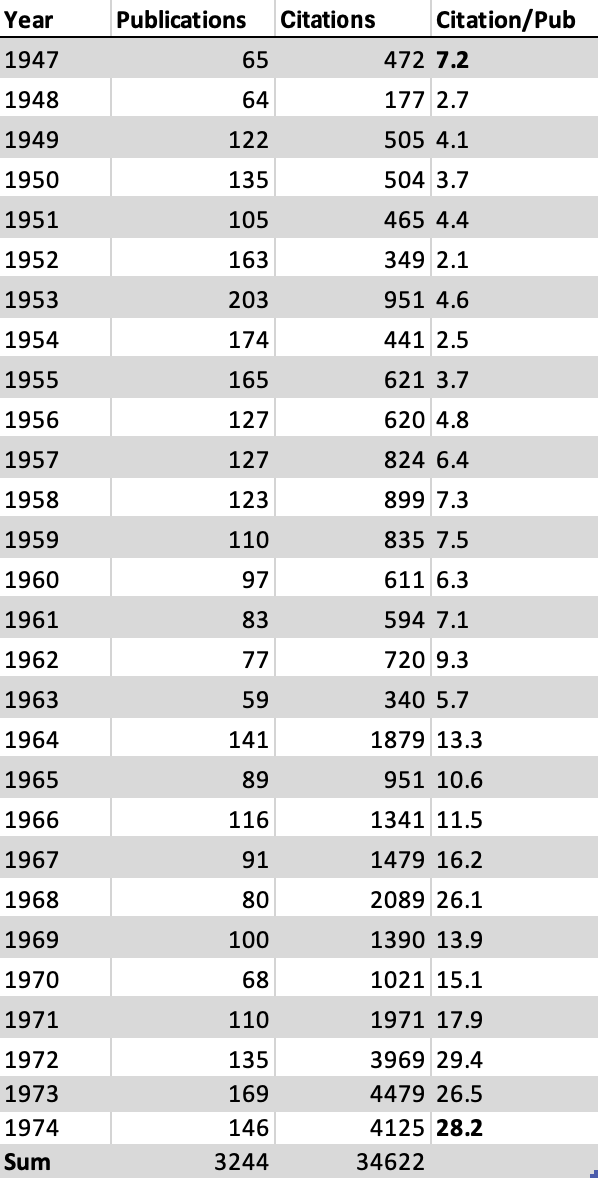


*Tab. S1 Trends in Publications (Original Papers) and Citations with Citation Quotient per Publication from 1947 to 1974 (last accessed 02.01.2024)*


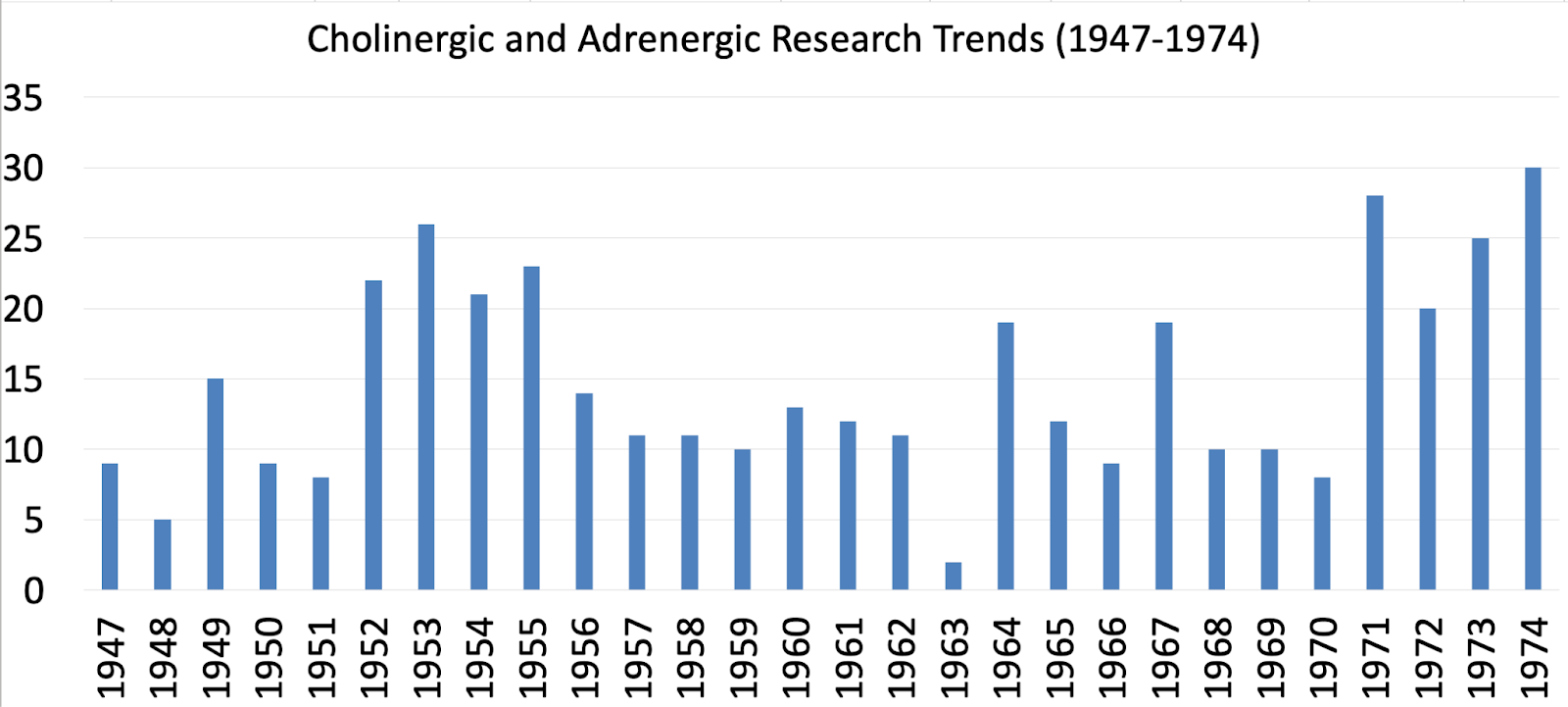


Number of publications

*Fig. S2 Trends in Cholinergic and Adrenergic System Research Publications (Original Papers) from 1947 to 1974*


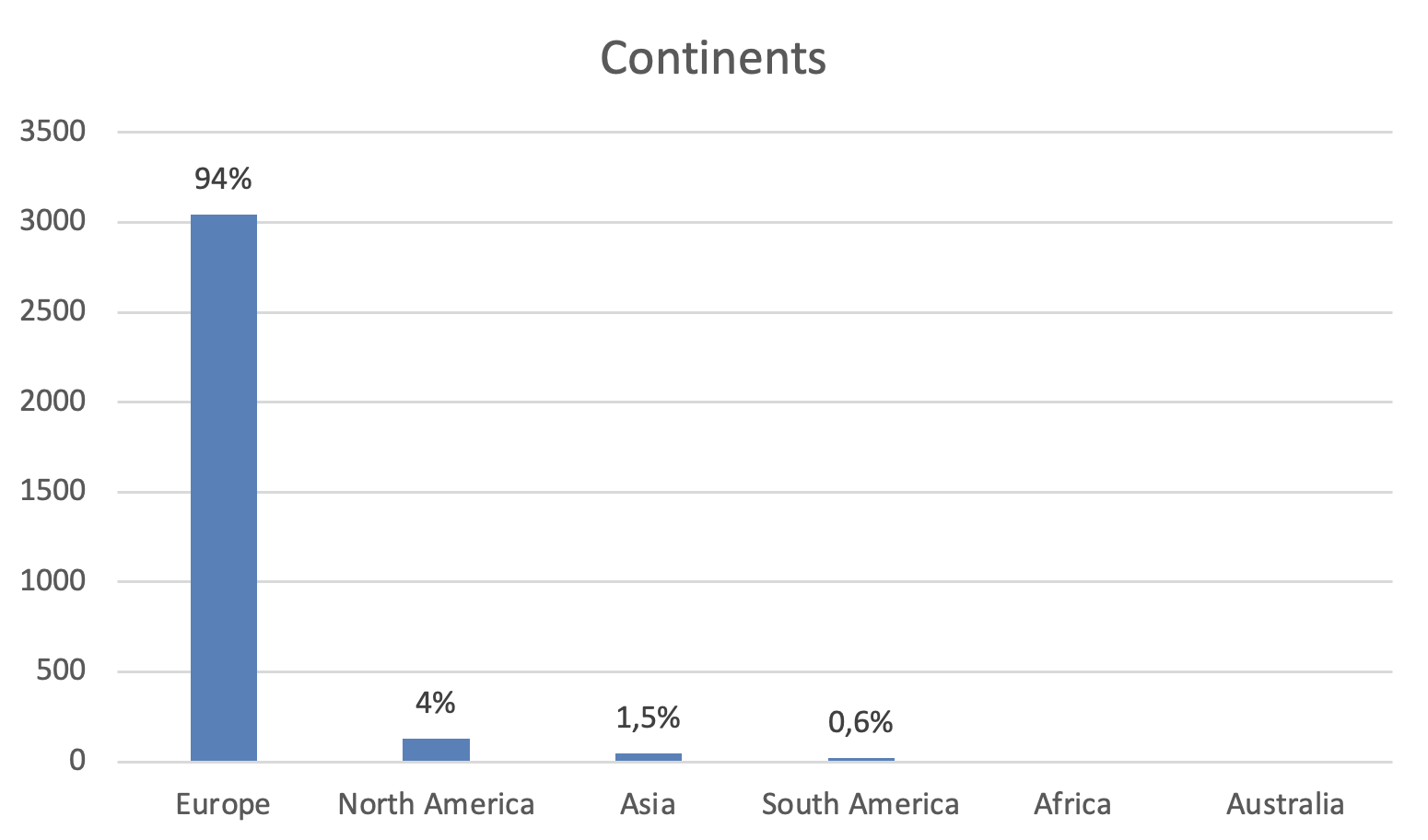

*Fig. S3 Percentage Share of Research Publications (Original Papers) by Continent from 1947 to 1974*

Number of publications


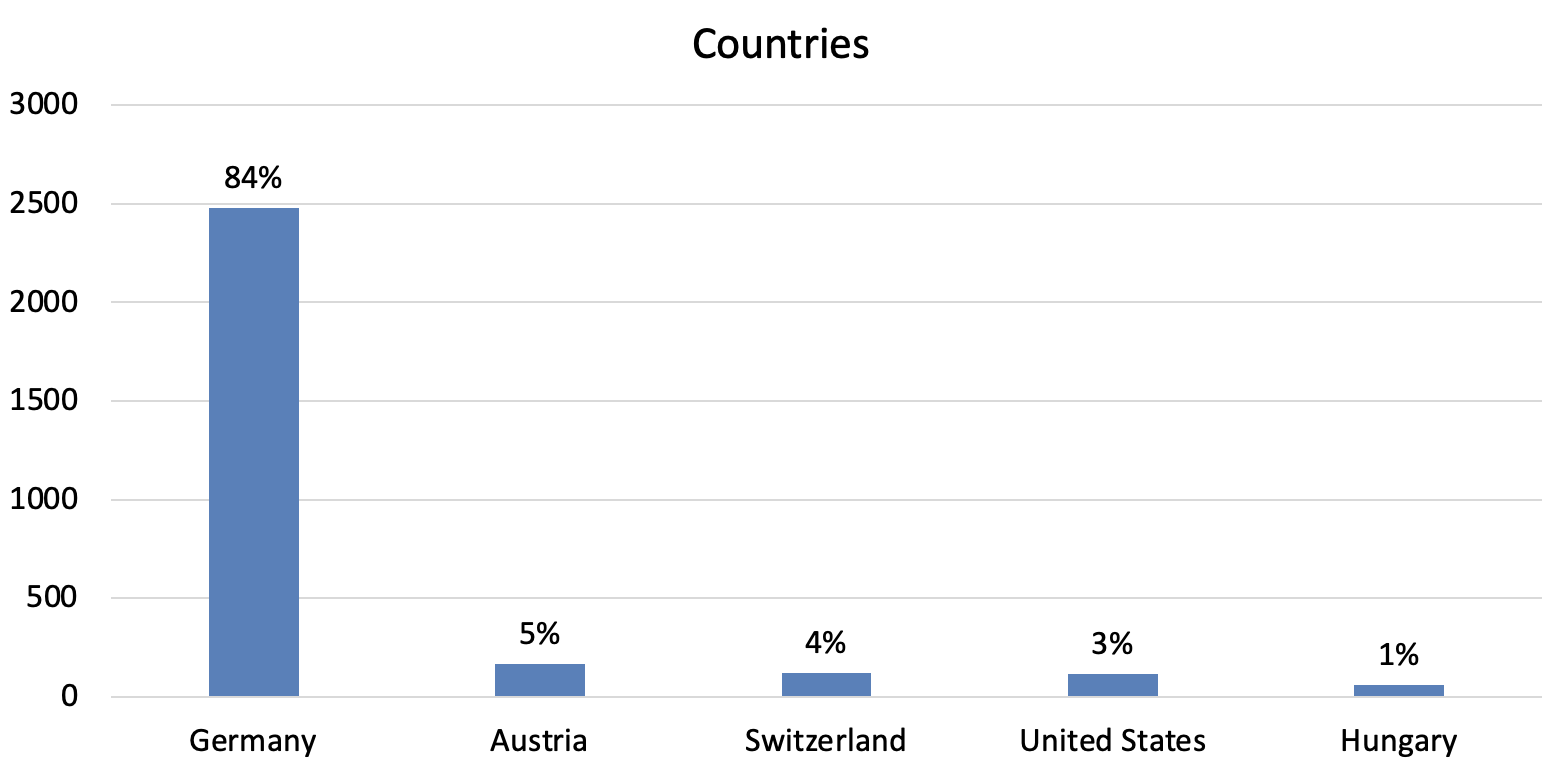

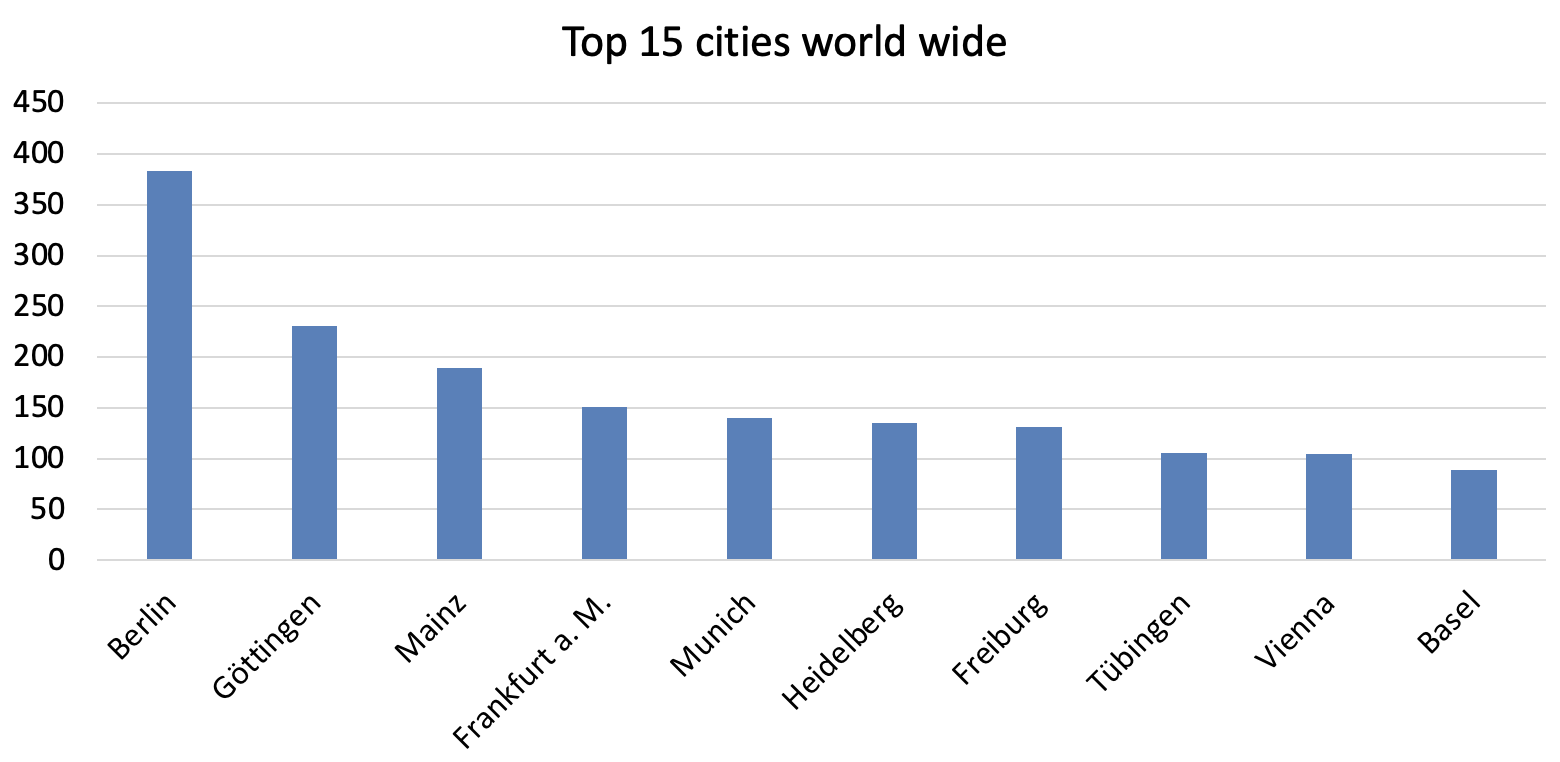


Number of publications

Number of publications

*Fig. S4 Top 5 Distribution of Research Publications (Original Papers) by Country from 1947 to 1974 Fig. S5 Ranking of Top 15 Cities Worldwide by Number of Research Publications (Original Papers) from 1947 to 1974*

| **Authors** | **Publications** | **Affiliations** | **Research focus** | **Language** |
| --- | --- | --- | --- | --- |
| Manfred Kiese  (1910-1983) | 47 | Graduated with a doctorate in 1935 in Berlin under W. Heubner Completed his habilitation there in 1939 Became the head of the pharmacological laboratory at the University Clinic in Kiel in 1947 Held a position in Munich from 1961 to 1980 | Pharmacodynamics and pharmacokinetics of methemoglobin and hemoglobin | Published 10 articles in English, starting in 1963 |
| Gerhard Zetler  (1921-2007) | 29 | Began his scientific career in 1949 at Christian-Albrecht University in Kiel, active until 1964 Completed his habilitation in 1954 and was appointed as a professor in 1959 Post-1964, transitioned to the Institute for Experimental and Clinical Pharmacology and Toxicology at the University Hospital Schleswig-Holstein in Lübeck Served as the first director of the Institute of Pharmacology and Toxicology there Published 18 articles in Kiel from 1951 to 1964 and 11 articles in Lübeck from 1967 to 1974 | Substance P | Since 1971, 6 publications in English |
| Gustav Kuschinsky (1904-1992) | 29 | Initiated career under Trendelenburg in Berlin, 1929 Habilitated with Heubner, then to Shanghai as a professor, 1934  Full professor in Prague, 1939 Directed Mainz Pharmacological Institute, 1946-1972 | Cholinergic and adrenergic systems | none in english, last publication in 1968 |
| Ernst Habermann (1926-2001) | 27 | 1954-1966: University of Würzburg, Institute of Pharmacology and Toxicology 1967-1974: Justus Liebig University Giessen, Institute of Pharmacology, Academy of Medical Research and Education | Toxicology - Honeybee venom | From 1971 to 1974, a total of ten articles were published in English |
| Peter Holtz (1902-1970) | 26 | 1938-1952: Worked at the Pharmacological Institute, University of Rostock. Held professorship in 1945, then became director. 1953: Joined Goethe University Frankfurt as Pharmacology Chair. Stayed until 1968 | Cholinergic and adrenergic systems: Noradrenalin | none in english |

*Tab. S2 Authors, Publication Counts (Original Papers), Affiliations, Research Focus, and Language of Publication (Philippu A. 2004, S. 48, 61, 99, 138-139, 344, 346, 401, 406, 444)*


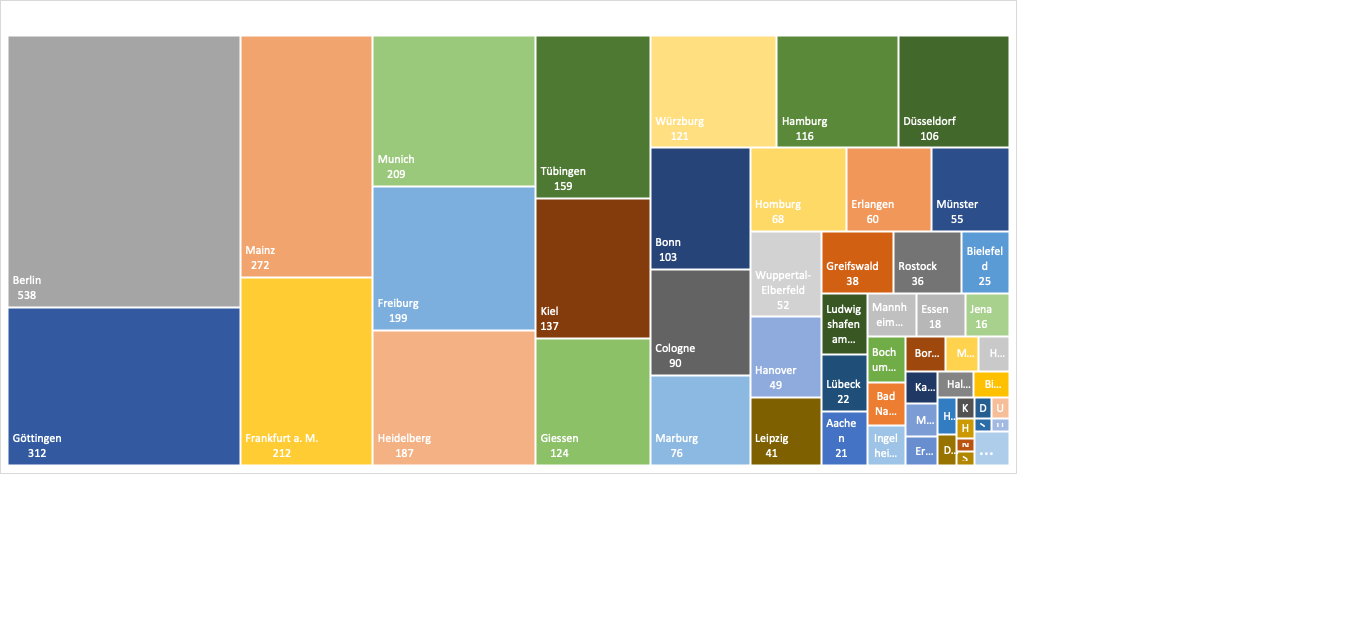


*Fig. S7 Tree map Visualization of Publication Distribution (Original Papers) across German Cities*

## References

Philippu A (2004) Geschichte und Wirken der pharmakologischen, klinisch-pharmakologischen und toxikologischen Institute im deutschsprachigen Raum. 1. Berenkamp, Innsbruck
